# Enzymatic Bio-Oxidation of Ethyl Hydroxyethyl Cellulose and UV-Assisted Cross-Linking with Chitosan for Enhanced Aqueous Stability in Biomaterial Production

**DOI:** 10.1021/acsomega.6c08601

**Published:** 2026-10-19

**Authors:** Beatriz Fernanda da Silva, Lidiane Meier, Ismael Casagrande Bellettini, Karina Cesca, Ana Paula Serafini Immich, Patrícia Bulegon Brondani

**Affiliations:** † Departamento de Ciências Exatas e Educação, Centro Tecnológico, de Ciências Exatas e Educação, 28117 Universidade Federal de Santa Catarina, Rua Marechal Rondon, 880 - Salto do Norte, Blumenau, Santa Catarina 89065-200, Brasil; ‡ Departamento de Engenharia Química e Engenharia de Alimentos, Centro Tecnológico, Córrego Grande, Florianópolis, Santa Catarina 88040-900, Brasil; § Departamento de Química, Centro de Ciências Físicas e Matemáticas, Universidade Federal de Santa Catarina, Rua Roberto Sampaio Gonzaga, s/n - Trindade, Florianópolis, Santa Catarina 88040-380, Brasil

## Abstract

Ethyl hydroxyethyl cellulose (EHEC) is a promising sustainable biomaterial, but its high hydrophilicity limits its use in moisture-resistant applications. Conventional cross-linking to improve aqueous resistance often requires toxic reagents, organic solvents, or high temperatures. Here, we developed a green cross-linking method by enzymatically oxidizing EHEC with laccase/TEMPO (2,2,6,6-tetramethylpiperidin-1-oxyl), followed by UV-induced cross-linking with chitosan at room temperature, yielding a robust macromolecular network. The optimal conditions (4 h oxidation, 50 wt % chitosan, 24 h cross-linking) yielded prolonged aqueous-stable films that maintained structural stability for 3–5 months in aqueous media, depending on pH. Compared to purified EHEC, the biomaterial exhibited enhanced thermal behavior, a 44% increase in elastic modulus (775.6 ± 172.8 MPa), reduced hydrophilicity (75.2 ± 0.7° contact angle, compared to 56.5 ± 0.6° from the original EHEC film), and no release of toxic compounds in cytotoxic assays with L929 fibroblast cells. This methodology is tunable and efficiently produces stable, sustainable EHEC-based films, demonstrating significant promise for advanced aqueous applications and active-ingredient encapsulation.

## Graphical abstract

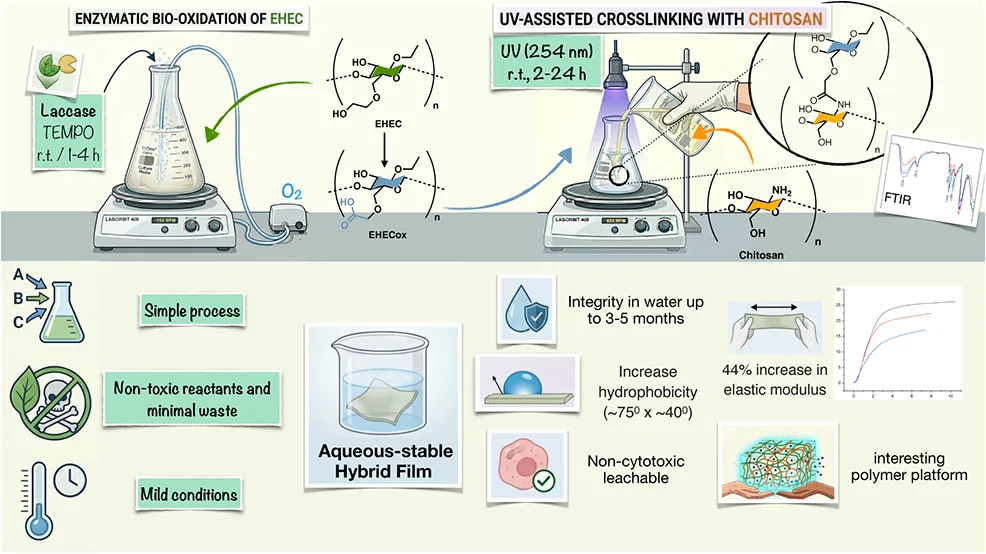

## 1 Introduction

The search for sustainable solutions has driven the development of new polymeric materials that combine functionality and biodegradability. The goal is to meet the demands of a market increasingly committed to sustainability and reducing environmental impacts, without compromising the technical performance of the materials.^1–4^ Due to its abundance, renewability, and biodegradability, researchers widely exploit cellulose and its derivatives in the production of sustainable materials.^5^

Among cellulose derivatives, the cost-effective ethylhydroxyethylcellulose (EHEC) is a highly promising material. EHEC exhibits ethyl and hydroxyethyl substitutions along the cellulosic backbone, imparting amphiphilic character, biocompatibility, low toxicity, and excellent film-forming capacity. These properties, combined with low cost, make it an interesting polymer for a range of industrial and research applications, including use as a thickener and water-retention agent in pharmaceutical formulations and in civil construction.^6–10^

Despite their advantageous structural properties, the high hydrophilicity of EHEC films leads to extreme water solubility, severely limiting their use in applications requiring structural integrity under high-humidity conditions, such as bandages and food packaging.^3,8,11,12^

To overcome the inherent water sensitivity of cellulose derivatives, researchers have extensively explored various physical and chemical modification strategies in recent years. Physical approaches often involve polymer blending to form polyelectrolyte complexes—such as combinations with chitosan, starch, or gelatin—or incorporating nanomaterials, including metal oxides, to restrict polymer chain mobility. Chemical modification, by contrast, remains a highly effective route to impart long-term dimensional stability by forming robust covalent three-dimensional networks. Recent advances in the sustainable chemical modification of related materials, such as carboxymethyl cellulose (CMC), include using green condensing agents such as citric acid to lead to ester bond formation or diamines in the presence of condensation agent (eg.: 4-(4,6-dimethoxy-1,3,5-triazin-2-yl)-4-methyl-morpholinium chloride—DMTMM) to construct amide bonds.^13–15^

Despite these greener innovations, conventional covalent cross-linking methods for cellulose derivatives still frequently rely on highly toxic synthetic bifunctional agents (such as glutaraldehyde, glyoxal, or epichlorohydrin) or require harsh reaction conditions. These reagents and processes can compromise the biological safety of the resulting material and create undesirable environmental liabilities.^16–19^

Based on these considerations, we investigated a covalent cross-linking route to produce water-insoluble EHEC-based films. As a first step, we investigated selective biocatalytic oxidation of EHEC mediated by the laccase/TEMPO (2,2,6,6-tetramethylpiperidine-1-oxyl) system. Laccases are multicopper oxidases that play a fundamental role in the bio-oxidation of cellulose-derived polymers. Using redox mediators, such as TEMPO, which facilitate electron transfer between laccase and the target substrate, can enhance the efficiency of this conversion.^20,21^ Although no specific studies have used this system to oxidize EHEC, it is reasonable to infer that it works similarly to the oxidation of other cellulose derivatives. In contrast to aggressive chemical oxidations, this system operates under mild conditions, converting primary hydroxyl groups into electrophilic carboxylic groups (COOH) while preserving the integrity of the carbohydrate chain.^22–24^

In a subsequent step, this study investigated the COOH-reactive sites introduced into the EHEC structure to promote cross-linking with chitosan, an aminopolysaccharide rich in nucleophilic groups (−NH_2_) and widely used as a matrix for new materials. This strategy aims to exploit macromolecular stabilization, thereby improving the material’s mechanical strength without additives, high temperatures, or organic solvents.^4,24–27^ Chitosan possesses notable characteristics, including solubility in acidic media, high chemical reactivity, and the ability to interact with a wide range of molecules and surfaces. Researchers widely use chitosan films in wound dressings because of their flexibility, biocompatibility, and ability to incorporate therapeutic agents, such as antibiotics and growth factors, that promote accelerated, safe recovery.^28^

The novelty of this study lies in the synergistic combination of these reaction pathways—enzymatic biocatalysis coupled with polysaccharide-mediated macromolecular cross-linking. Thus, this study aims not only to optimize the properties of EHEC but also to expand its potential for industrial applications requiring aqueous stability and sustainability, thereby contributing environmentally responsible alternatives to conventional polymers.

## 2 Experimental Part

### 2.1 Materials

Chemical reactants, enzymes and cells included: EHEC Bermocoll E 230FQ (Akzo Nobel, Sweden), with a degree of substitution of 0.9–1.0 for ethyl groups and 1.9–2.2 for ethylene oxide per anhydroglucose unit; laccase Novoprime Base 286; the redox mediator 2,2,6,6-tetramethylpiperidine-1-oxyl (TEMPO) (Sigma-Aldrich); chitosan (Sigma-Aldrich, medium molecular weight, degree of deacetylation 76%); DMEM (Dulbecco’s Modified Eagle’s Medium); 10% fetal bovine serum (FBS) (Gibco, Brazil); 1% penicillin–streptomycin (pen/strep); PBS (phosphate-buffered saline) (Gibco, Brazil); sodium carbonate (Sigma-Aldrich); L929 cells (murine fibroblast cell line) (Thermo Scientific); MTS [3-(4,5-dimethylthiazol-2-yl)-5-(3-carboxymethoxyphenyl)-2-(4-sulfophenyl)-2H-tetrazolium] (Promega Biotecnologia do Brasil, Brazil);

### 2.2 EHEC Bio-Oxidation

We solubilized EHEC in distilled water (pH 6) by magnetic stirring for 24 h. Then, we added 17.5 mg of TEMPO and 15 mg of laccase (Novoprime Base 286) to the solution. We carried out the reaction for 1–4 h under continuous orbital shaking (orbital shaker KLINE-KJ201BS, 150 rpm) at 30 °C and aeration (air compressor OceanMax, 3 L min^–1^). At the end of the reaction, we centrifuged the samples (centrifuge V4000-1215, 4000 rpm, 15 min) to remove the enzyme and any remaining residues. Finally, we transferred the resulting solutions to Petri dishes and dried them in an oven at 50 °C for approximately 5 days, yielding bio-oxidized EHEC (EHECox) as an opaque, yellowish film.

### 2.3 One-Pot Cross-Linking of EHECox with Chitosan

The cross-linking reaction was carried out in a one-pot system in a single step, without intermediate isolation of reagents. To prepare the films, a chitosan solution was first obtained by dissolving 250 mg of chitosan in 25 mL of a solution containing 0.16 mL of acetic acid, then magnetically stirred at 50 °C until complete solubilization. This solution was then added to 25 mL of previously prepared EHECox at a 2:1 (EHECox: chitosan) ratio, and the mixture was stirred (150 rpm) for 1–24 h under UV irradiation (254 nm, 30 W/m^2^) at room temperature. In addition, as a control, the same procedures were applied using nonoxidized EHEC. Samples of bio-oxidized EHEC and chitosan, separately exposed to UV, were also used as controls. After each exposure time, the samples were transferred to Petri dishes and dried in an oven at 50 °C until the film was fully formed.

### 2.4 Film Cytotoxicity Test

The cytotoxic potential and cytocompatibility of the EHECox4Q24 polymeric film was evaluated using an *in vitro* cytotoxicity assay with L929 cells, in accordance with ISO 10993-5 (ISO/EN10993-5, 2009). According to the standard (ISO 10993-5), materials presenting cell viability above 70% are considered noncytotoxic. Cytotoxicity was assessed using extract and direct-contact assays. For extract preparation, films were UV-sterilized (30 min per side) and incubated in 200 μL of Dulbecco’s Modified Eagle Medium (DMEM) for 24 h at 37 °C. The extracts were collected and diluted from 0–100%. The effect of a PBS prewashing step of the film (2 h) was also evaluated. L929 cells were cultured in DMEM supplemented with 10% FBS (Fetal Bovine Serum) and 1% penicillin-streptomycin and seeded at 1 × 10^4^ cells/well. After 24 h, cells were exposed to the extracts and incubated for 24 h. Cell viability was determined using the MTS assay, with absorbance measured at 490 nm.

Direct contact tests were performed only on thicker films, as thinner samples dissolved upon hydration. Films were hydrated in DMEM for 24 h, dried for 2 h, and seeded with 3 × 10^4^ cells in 10 μL of medium. After 4 h of cell adhesion, 200 μL of DMEM medium was added to each well, and the cultures were incubated for an additional 24 or 72 h. Metabolic activity was evaluated by the MTS assay after transferring the films to a new plate. Statistical analysis was performed using ANOVA followed by Tukey’s test (*p* < 0.05), and results were expressed as mean ± standard deviation.

### 2.5 Film Biodegradability Test

The biodegradability test was performed using the optimized polymeric film EHECox4Q24 (EHEC oxidized for 4 h and cross-linked with chitosan under UV irradiation for 24 h). Biodegradation assays were carried out in simulated soil prepared from equal proportions of fertile low-clay soil, cured horse manure, and coarse sand (10–40 mesh). The mixture was sieved and matured for approximately 3 months in accordance with ASTM G160-03.

Tests were conducted in triplicate to ensure reproducibility. Film samples (2.0 × 2.0 cm) were individually buried in separate containers under controlled, homogeneous conditions. After 2 and 4 months, the samples were removed, washed with distilled water, and dried at room temperature. The degree of disintegration was then evaluated based on mass loss over time.

### 2.6 Characterization of EHEC/Chitosan Films

The EHEC/chitosan films obtained under different bio-oxidation and cross-linking conditions were characterized through physicochemical, structural, thermal, and mechanical analyses. The films were evaluated for structural integrity, water stability, solubility, swelling behavior, wettability, and degree of oxidation. Thermal properties were investigated by thermogravimetric analysis (TGA), using TGA 8000 (PerkinElmer). The samples were heated from 30 to 700 °C under a continuous argon flow at 10 °C/min. The samples were packaged in sealed aluminum capsules and subjected to an inert atmosphere (continuous nitrogen flow). The test was conducted at 10 °C/min from 0 to 200 °C, followed by cooling at the same rate. Chemical and structural characterization was performed using FTIR-ATR (PerkinElmer Frontier) and X-ray diffraction (XRD; Bruker D6 Phaser, equipped with a Cu Kα radiation source (λ = 1.5406 Å) operating at 40 kV and 15 mA). For the FTIR-ATR analysis, the data were normalized by the maximum value of each series, yielding relative values between 0 and 1. FTIR deconvolution technique, where the Gaussian-Lorentz function is adapted to the Origin Lab software. Scanning electron microscopy (SEM) images were obtained at 1000× and 2500× using a JEOL JCM-7000 Benchtop scanning electron microscope. The optimized film was also assessed for stability across different pH conditions, gravimetric behavior in an aqueous medium, and mechanical properties via tensile strength tests.

The analyses were conducted using a LUCA-150 conductometer. The degree of oxidation (DO) was determined by conductometric titration. The DO was calculated according to Habibi, Chanzy, and Vignon (2006) (eq 1).^29^

1 $$DO=\frac{162\times C\times \left(V_{2}-V_{1}\right)}{w-19\frac{g}{mol}\times C\times \left(V_{2}-V_{1}\right)}$$

All quantitative experiments, including water contact angle measurements, DO, and swelling degree, were performed in triplicate. The mechanical properties were evaluated five times for each sample (n = 5). Statistical analysis was conducted using one-way analysis of variance (ANOVA), and sample groups were differentiated using Tukey’s test, with significance at p = 0.05. Data were processed using RStudio v1.4.1717. Results are expressed as mean ± standard deviation.

## 3 Results and Discussion

### 3.1 Bio-Oxidation of EHEC

To prepare the oxidized EHEC derivative (EHECox), we employed a laccase-catalyzed bio-oxidation using Novoprime Base 286 in the presence of TEMPO, with constant aeration and orbital agitation for 1–4 h. In this synergistic system, TEMPO acts as an electron-transfer mediator, while laccase promotes oxidation of primary hydroxyl groups in the polymer structure, converting them to carbonyl or carboxyl groups.^30^ Compared with the bio-oxidation of cellulose, which preferentially occurs at the primary hydroxyl group of C6 in the anhydroglucose unit,^31^ EHEC probably exhibits distinct behavior because the hydroxyethyl moiety becomes the most favorable site for oxidation (Scheme 1). Thus, the proposed oxidative pathway of EHEC leads to a new structural organization and promotes the formation of reactive sites that are important for subsequent steps, such as cross-linking with chitosan.

**Scheme 1:**
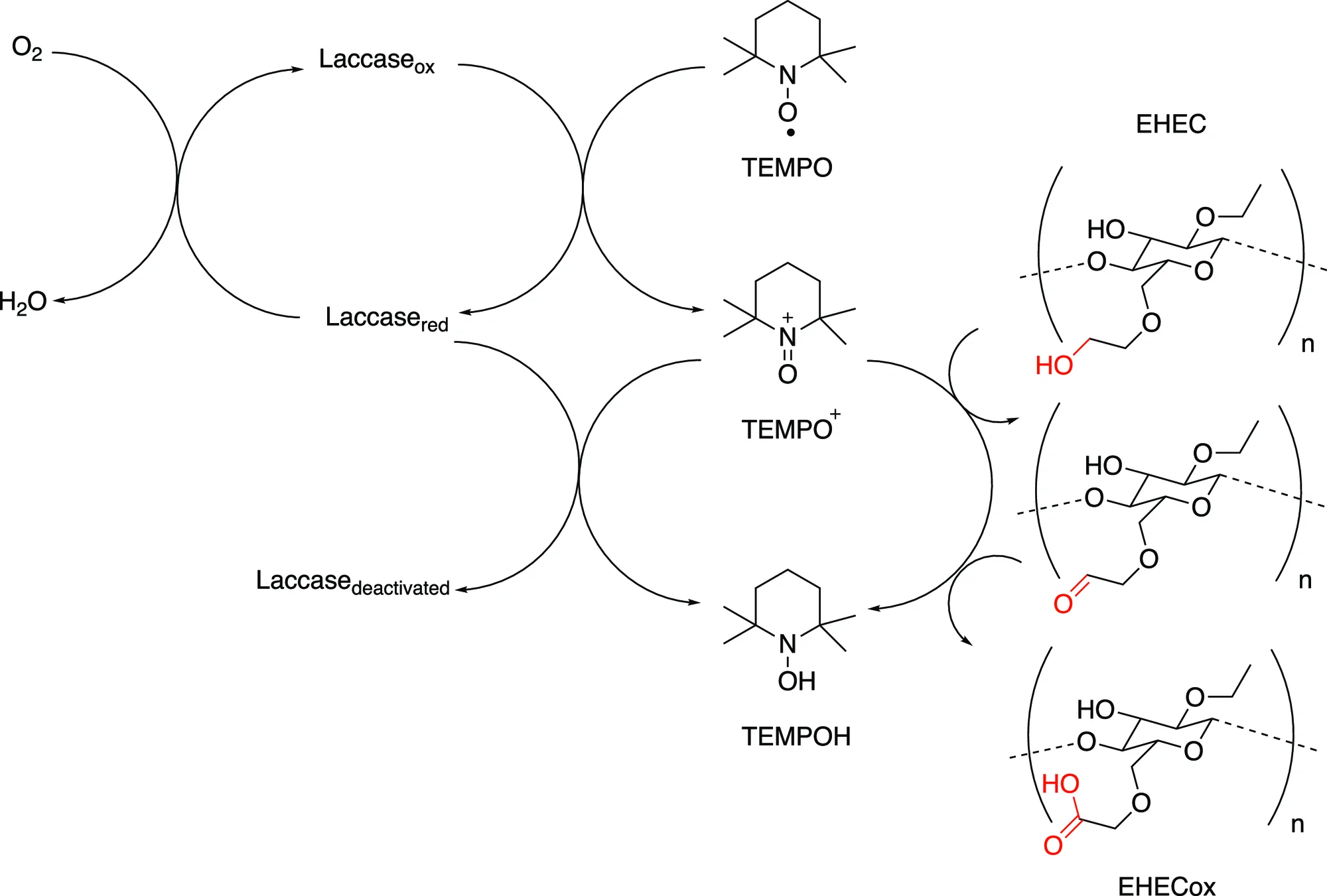
Mechanistic Proposition for EHEC Oxidation Using Laccase/TEMPO

After oxidation for 1–4 h, we dried the EHECox samples and measured their water contact angles. The resulting EHECox films exhibited low structural stability in the presence of water droplets and an average water contact angle of 40.2 ± 0.8° (*p* > 0.05) (Figure 1). However, we observed greater wettability than that of unoxidized EHEC (56.5 ± 0.6°), probably due to exposed −COOH groups on the surface of the EHECox films, indicating that isolated oxidation does not increase hydrophobicity.^32^

**Figure 1:**
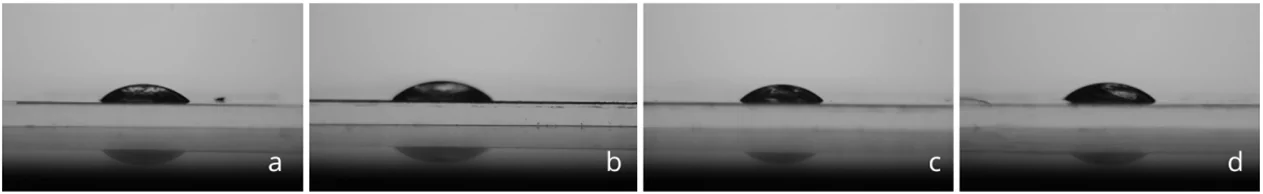
Water contact angle for EHEC films bio-oxidized during a) 1 h, b) 2 h, c) 3 h, and d) 4 h reaction.

The FTIR spectra of the EHECox (1–4 h oxidation) samples were compared with those of EHEC without oxidation (Figure 2), displaying common characteristic bands associated with polysaccharides. The broad absorption at 3420 cm^–1^ is assigned to the O–H stretching, indicating hydrogen bonds and the possible presence of water. The band at 2872 cm^–1^ is attributed to the C–H stretching of methyl and methylene groups, while the band at 1653 cm^–1^ is associated with the in-plane angular deformation of adsorbed water. At 1351 cm^–1^, C–H or C–N deformation, and at 1041 cm^–1^, the C–O–C and C–O stretching are characteristic of glycosidic bonds. When comparing the FTIR spectra of the bio-oxidized samples at 1–4 h, no significant variations in spectral bands were observed, and the profiles were similar to those of the unoxidized EHEC. This behavior is common when dealing with the mild oxidation of cellulose-derived polymers, as the relatively low concentration of newly formed carboxylic groups yields a weak C=O stretching signal that is easily obscured by the strong overlapping band of adsorbed water at ∼1650 cm^–1^.^24,33^

**Figure 2:**
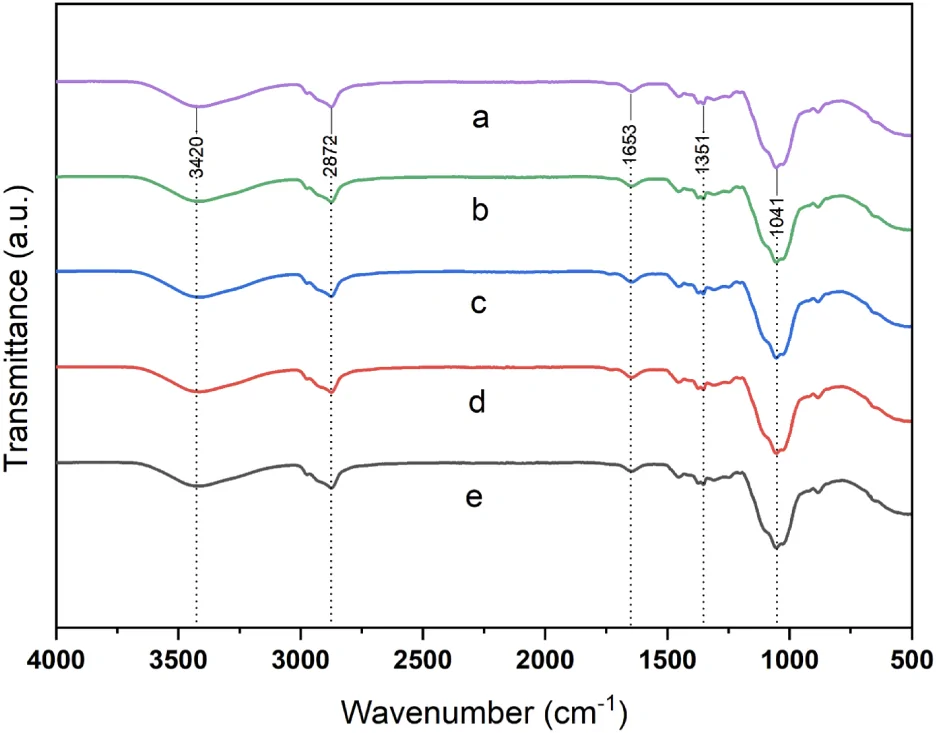
FTIR spectra of a) unoxidized EHEC and EHEC oxidized during b) 1 h, c) 2 h, d) 3 h, and e) 4 h.

Since we did not find significant differences across oxidation times in the analyses, we used 1 h for subsequent tests.

### 3.2 Preliminary Tests of EHECox Cross-Linking with Chitosan

Converting −CH_2_OH groups to carbonylated groups during the bio-oxidation step increases the polymer’s functionality, favoring cross-linking between the electrophilic groups of EHECox and the nucleophilic groups of chitosan, possibly resulting in stable three-dimensional networks.^34,35^

Under fixed bio-oxidation conditions (1 h), we applied three distinct approaches to promote cross-linking between EHECox and chitosan, enabling comparison of chemical strategies and identification of the most efficient alternative for forming insoluble, mechanically stable polymeric films. We evaluated three cross-linking strategies: (I) direct mixing at 50 °C, (II) EDC/DMAP-mediated coupling at 50 °C, and (III) direct mixing under UV light (254 nm) irradiation at room temperature. For all methods, we combined separately prepared EHECox and chitosan solutions (50 mL final volume, pH ∼5) and stirred them for 2 h. In strategy II, we used EDC and DMAP to activate the −COOH groups in the EHECox structure, then dialyzed to remove residual reagents.^36^ Whereas we directly oven-dried the samples from strategies I and III at 50 °C.

We performed a preliminary evaluation of the three cross-linking methods to select the most efficient approach for further investigation. This comparative study focused on aqueous solubility resistance (measured by mass loss), the maintenance of visual integrity, and FTIR analysis before and after a 48-h submersion period.

In approach (I), the film appeared translucent and uniform. However, after water submersion, we observed contraction, curling, and stiffening. After 48 h of submersion in water, mass loss was 25.0 ± 2.0 mg. For (II), the resulting film exhibited a smooth, translucent surface, but after 48 h of submersion in water, it developed deformations and tears, with a mass loss of 35.0 ± 1.0 mg. In contrast, method (III) produced homogeneous, thick films that, after 48 h in water, maintained their shape and structure, with a mass loss of 18.1 ± 2.2 mg.

Its FTIR spectrum (Figure 3c) showed higher band intensities than those of the other approaches, even after 48 h of water immersion, indicating intact functional groups and structural organization with no signs of degradation. The main bands observed correspond to the O–H and N–H stretches (∼3337 cm^–1^), the aliphatic C–H stretch (∼2889 cm^–1^), the characteristic C=O of amides (∼1649 cm^–1^), the Amide II band (∼1559 cm^–1^), and the C–H or C–N deformation (∼1411 cm^–1^).

**Figure 3:**
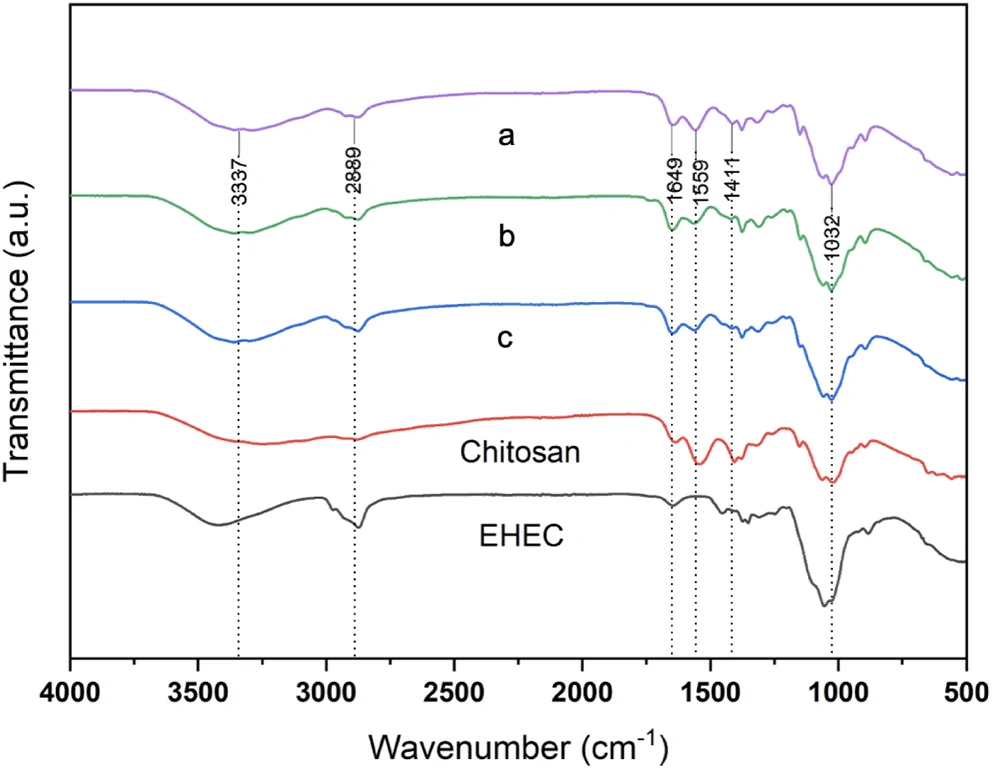
FTIR spectra of films obtained by three cross-linking methodologies, after immersion in water for 48 h, compared to EHEC and Chitosan starting materials. a) EHECox and chitosan at room temperature using UV light irradiation, b) EHECox and chitosan at 50 °C using DMAP/EDC, c) EHECox and chitosan at 50 °C.

Furthermore, UV light promoted effective cross-linking of polymeric macromolecules without additional reagents, making the technique efficient and environmentally sustainable for systems that employ polysaccharides and other natural biopolymers.

The photoirradiation of chitosan, particularly with UV–C (254 nm), has been widely studied in the literature, especially regarding photodegradation and changes in the properties of chitosan films and blends. Its impact on chitosan bioactivity depends heavily on the exposure conditions. Recent studies show that controlled UV irradiation alters the macromolecular structure but can preserve or even modulate specific bioactivities—such as antimicrobial or elicitor functions—by generating lower-molecular-weight functional fragments. While photoexposure can lead to chitosan chain scission (degradation), it can also trigger other reactions, depending on the reactive species present in the reaction medium. Although the use of chitosan in a context similar to that of the present study has not been explored, it is known that chitosans with a higher degree of deacetylation photodegrade more readily and that changes—particularly in the FTIR bands associated with O–H and NH_2_ groups—are observed upon irradiation for up to 24 h. Therefore, probably the UV–C irradiation directly initiates photochemical processes by generating localized reactive species, such as radical cations ${(\mathrm{R‐NH}}_{2}^{•+})$ or radicals (R-NH^•^).^37^

The reaction between primary amino groups and carboxylic acids or their derivatives, leading to amide formation via radical reactions, is already known. In these cases, the radical cation formed at the amino group reacts with the carboxyl group, forming the amide and eliminating water—formed from the hydroxyl radical lost by the carboxylic acid and the proton lost by the nitrogen-containing moiety.^38,39^ Although the radical pathway is chemically plausible and supported by literature precedents, the mechanism outlined in Scheme 2 is strictly postulatory.^37,40–42^

**Scheme 2:**
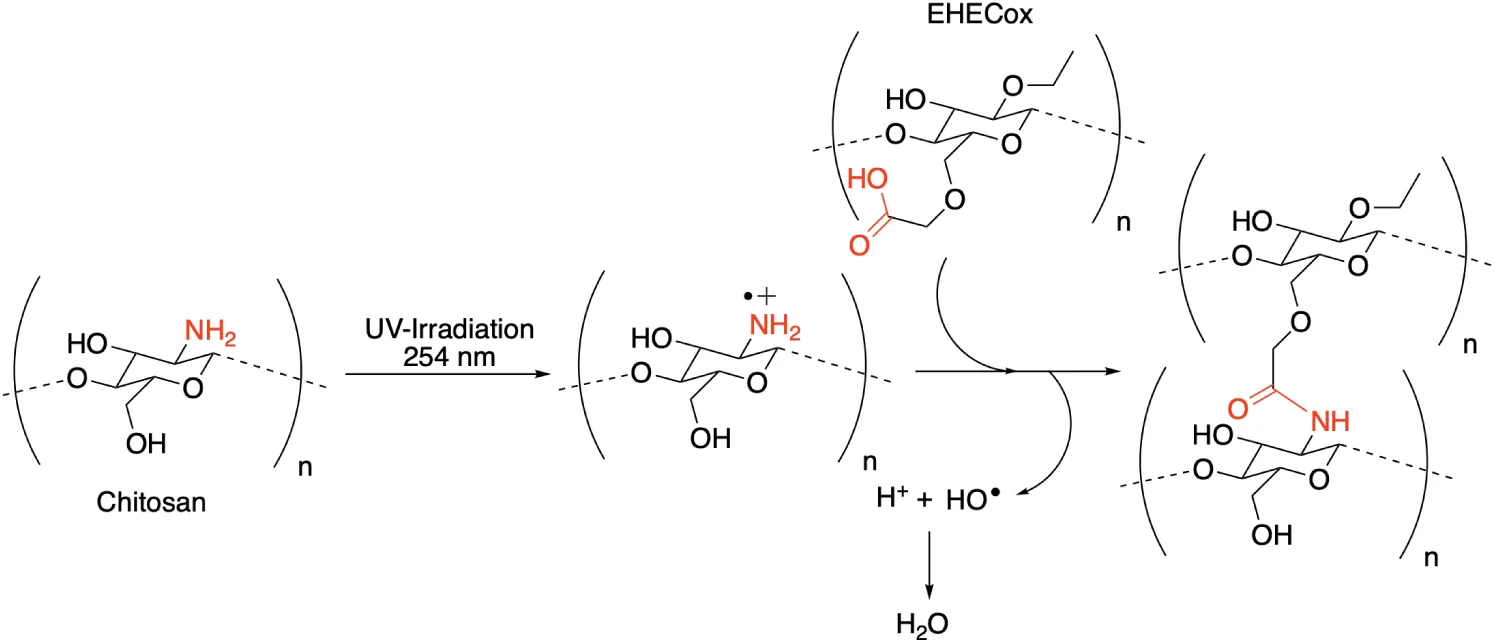
Plausible Reaction Pathway and Proposed Mechanism for the UV-Assisted Cross-Linking between EHECox and Chitosan

### 3.3 Study of Cross-Linked EHECox-Chitosan Mediated by UV Light

To elucidate the effects of EHEC oxidation time and UV light exposure on the properties of the resulting films, we conducted a series of tests and preliminary water submersion tests. The experiments were divided into four control and test groups: (IV) unoxidized EHEC exposed to UV light for 2 h; (V) EHECox (1–4 h oxidation) with or without 2 h of UV exposure; (VI) unoxidized EHEC/chitosan blend (2:1 proportion), with or without 2 h of UV irradiation; and (VII) EHECox (1–4 h oxidation)/chitosan blend (2:1 proportion) exposed to UV light for 2 h.

Solubility tests revealed that UV irradiation alone does not confer aqueous stability on pure EHEC films (group IV), as they solubilized within 40 min, matching the behavior of nonirradiated EHEC films. Similarly, pure EHECox films (group V) solubilized after 2 h, regardless of the oxidation time or UV exposure. Adding chitosan to unoxidized EHEC (group VI) significantly delayed solubilization to 10 days, suggesting that strong intermolecular interactions—such as hydrogen bonding—between the polymers improve aqueous stability. In contrast, group VII films (EHECox + chitosan + UV irradiation) maintained structural integrity for up to 3 months (Supporting Information, Figure S1).

Overall, the results indicate that stability was severely compromised in the absence of COOH groups under identical conditions. This reliance on the prior presence of carboxyl groups demonstrates that simple physical blending under UV is insufficient to yield long-term aqueous stability, indicating that a chemical cross-linking process—likely involving covalent interchain coupling alongside dense cooperative hydrogen bonding—is triggered by UV exposure in the presence of −COOH and −NH_2_ groups.

We also compared FTIR spectra (Figure 4) of EHECox and chitosan with those of the film generated by cross-linking EHECox and chitosan under UV light. The FTIR spectra remained largely similar regardless of the oxidation time used to produce EHECox.

**Figure 4:**
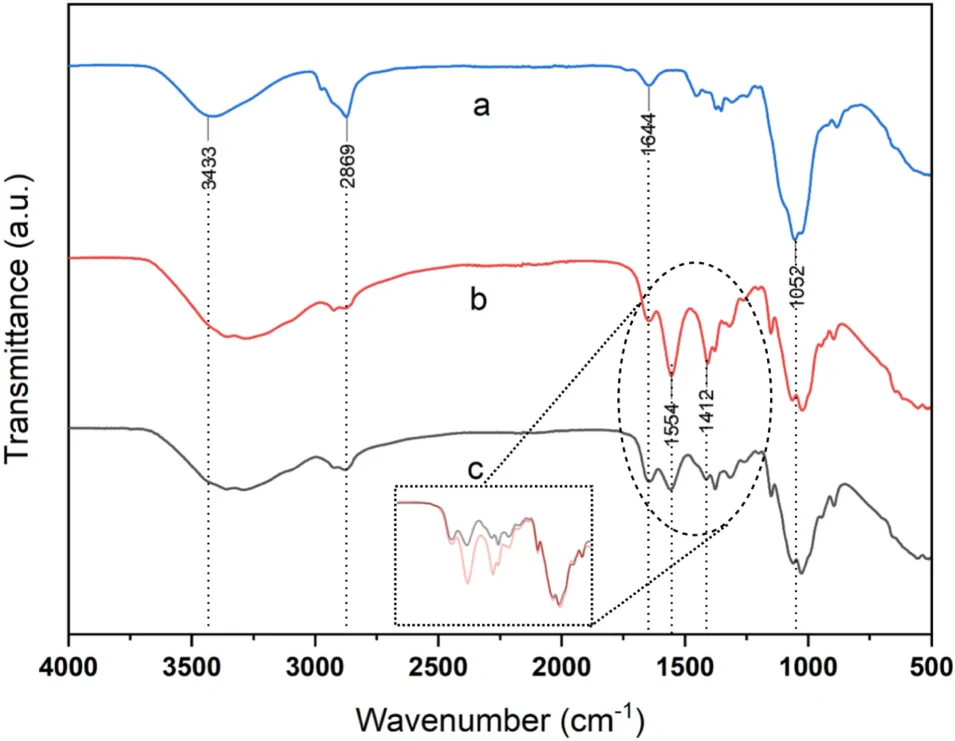
FTIR of films of a) EHECox, b) Chitosan, and c) EHECox cross-linked with chitosan under UV light irradiation.

Adding chitosan increases vibrational band intensities, especially in the O–H stretching region (3400 cm^–1^), due to hydrogen bonding in the cross-linked network. Compared with EHECox, the intensification of the N–H bending bands, along with the C–N stretching (∼1550 cm^–1^), is also noteworthy. Furthermore, the C=O stretching band at ∼1600 cm^–1^, present in Figure 4b and 4c but absent in Figure 4a, reinforces the structural alteration. These bands remain in the spectra even after washing the film with water for 24 h.

Surface and chemical characterizations further corroborated the cross-linking efficiency. Contact angle measurements for group VII films (59.2 ± 4.8°) indicated greater hydrophobicity than those for pure EHECox films (40.2 ± 0.8°; *p* < 0.05) (Figure 1). This reduction in surface polarity is consistent with the involvement and partial consumption or shielding of hydrophilic −NH_2_ and −COOH moieties during network assembly and potential covalent bonding, altering the surface energy. Progressive oxidation times did not significantly affect these values.^18^

Additionally, conductometric titration was employed to quantify the degree of oxidation (DO) of EHECox in group VII films (Supporting Information, Figure S2). The average DO was 0.17 ± 0.03 (CV = 16.3%). Although the 4 h oxidized sample exhibited the highest DO (0.21), the data dispersion suggests no statistically significant differences (*p* > 0.05) among the evaluated times, indicating that available functional groups on the EHEC surface may have become saturated within the first hour of the reaction.^29^

Overall, these findings demonstrate that UV irradiation effectively mediates the chemical cross-linking of EHECox and chitosan, yielding a highly stable polymeric network. While similar cross-linking reactions have been reported using agents such as carbodiimides and aziridines, this study highlights a novel UV-mediated interaction between chitosan and an oxidized cellulose derivative.^37^

#### 3.3.1 Evaluation of the Amount of Chitosan Used in the Cross-Linking Reaction with EHECox

We tested different chitosan proportions (15%, 25%, and 50% relative to EHECox mass), keeping the EHECox amount (500 mg) constant and the oxidation step at 1 h. Increasing chitosan concentration intensified the yellowish coloration of the films. When submerged in water, films with lower chitosan content (15% and 25%) swelled significantly and fragmented within 40 min, with the 15% sample completely dissolving after 53 days. In contrast, the 50% chitosan film demonstrated lower water absorption and superior stability, maintaining its structural integrity for 3 months (Supporting Information, Figure S3). This enhanced aqueous stability likely stems from a denser polymeric network, including hydrogen bonds and possibly covalent bonds.

The FTIR analyses showed intensified amide I (∼1650 cm^–1^) and II (∼1568 cm^–1^) bands in the sample containing 50% chitosan, even after washing, suggesting stronger interactions between the functional groups (Figure 5).

**Figure 5:**
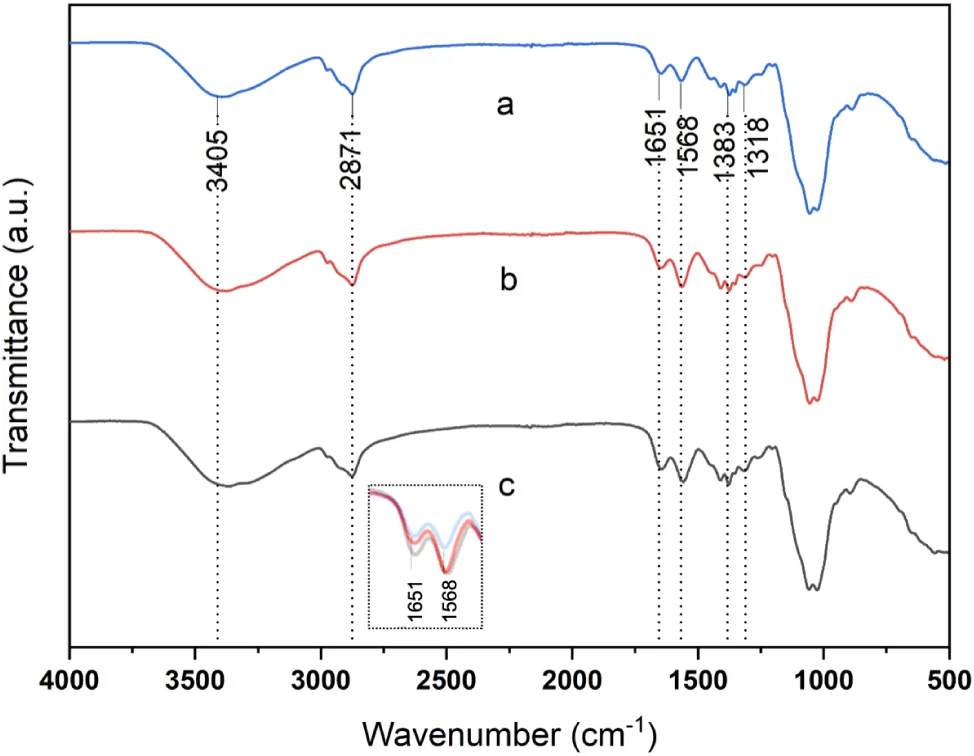
FTIR of the film with a) 15%, b) 25%, and c) 50% of chitosan.

Because the 15% and 25% chitosan formulations underwent macroscopic fragmentation and complete dissolution, continuous quantitative monitoring (e.g., precise long-term swelling or mechanical testing) was not viable. Therefore, the 50% chitosan formulation was selected not based on small numerical differences, but on its unique capacity to cross the threshold into full structural stability for up to 3 months.

#### 3.3.2 Optimization of UV Light-Mediated Cross-Linking Time

To optimize the cross-linking reaction time between EHECox and chitosan, while keeping the oxidation step at 1 h, we tested shorter cross-linking times (up to 90 min) and compared the results with those from 2 h. However, the resulting films were fragile and had lower aqueous stability. This result, although qualitative, indicates a temporal dependence in the cross-linking step under UV irradiation.

Despite the positive results obtained with films cross-linked from EHECox and chitosan for 2 h, FTIR analysis revealed that the intensities of the absorption bands varied across different regions of the same film, indicating unsatisfactory homogeneity. Therefore, we conducted a new set of tests, extending the cross-linking time under UV irradiation from 2 to 24 h. For this test, EHECox oxidized for 1 or 4 h was used for comparative purposes (Table 1).

**Table 1:** Sample Identification Codes for Films Generated by Oxidation of EHEC, Followed by Crosslinking with Chitosan under UV Light at Different Times

Sample code	Time of oxidation of EHEC (h)	Time of cross-linking with chitosan (h)
EHECox1C2	1	2
EHECox4C2	4	2
EHECox1C24	1	24
EHECox4C24	4	24

The analysis of the FTIR spectra reveals that the samples EHECox1C24 and EHECox4C24 (Figure 6b and 6c) exhibit strong bands in the region between 3374 and 1407 cm^–1^, indicating a significant increase in the density of functional groups and the structural organization of the polymer network when compared with EHECox4C2 (Figure 6a). We did not observe significant differences between the EHECox1C2 and EHECox4C2 spectra; only the latter are shown. Despite being subtle, EHECox4C24 (Figure 6c) showed intensified bands in the 1400–1650 cm^–1^ region compared to EHECox1C24, indicating that increasing the oxidation time, combined with prolonged cross-linking periods, influences the structural organization of the films. We likely did not observe differences in oxidation times when the cross-linking step was maintained at 2 h because there was insufficient time for all the COOH groups generated in the EHECox to react with the chitosan NH_2_ groups.

**Figure 6:**
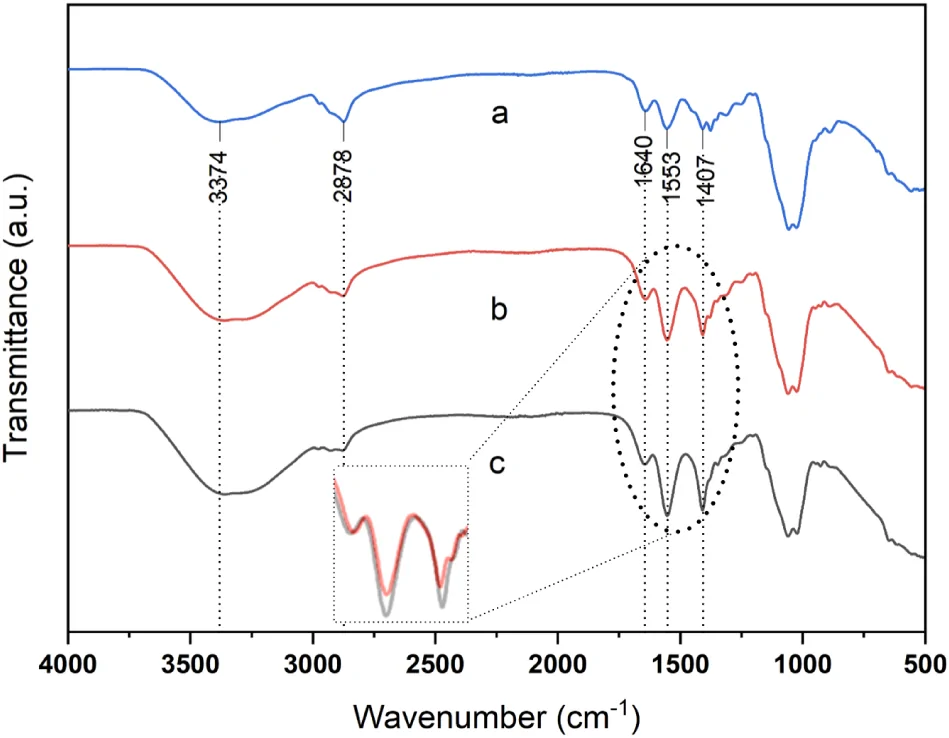
FTIR of EHEC and chitosan-based films: a) EHECox4C2, b) EHECox1C24 film, and c) EHECox4C24 film.

**Figure 7:**
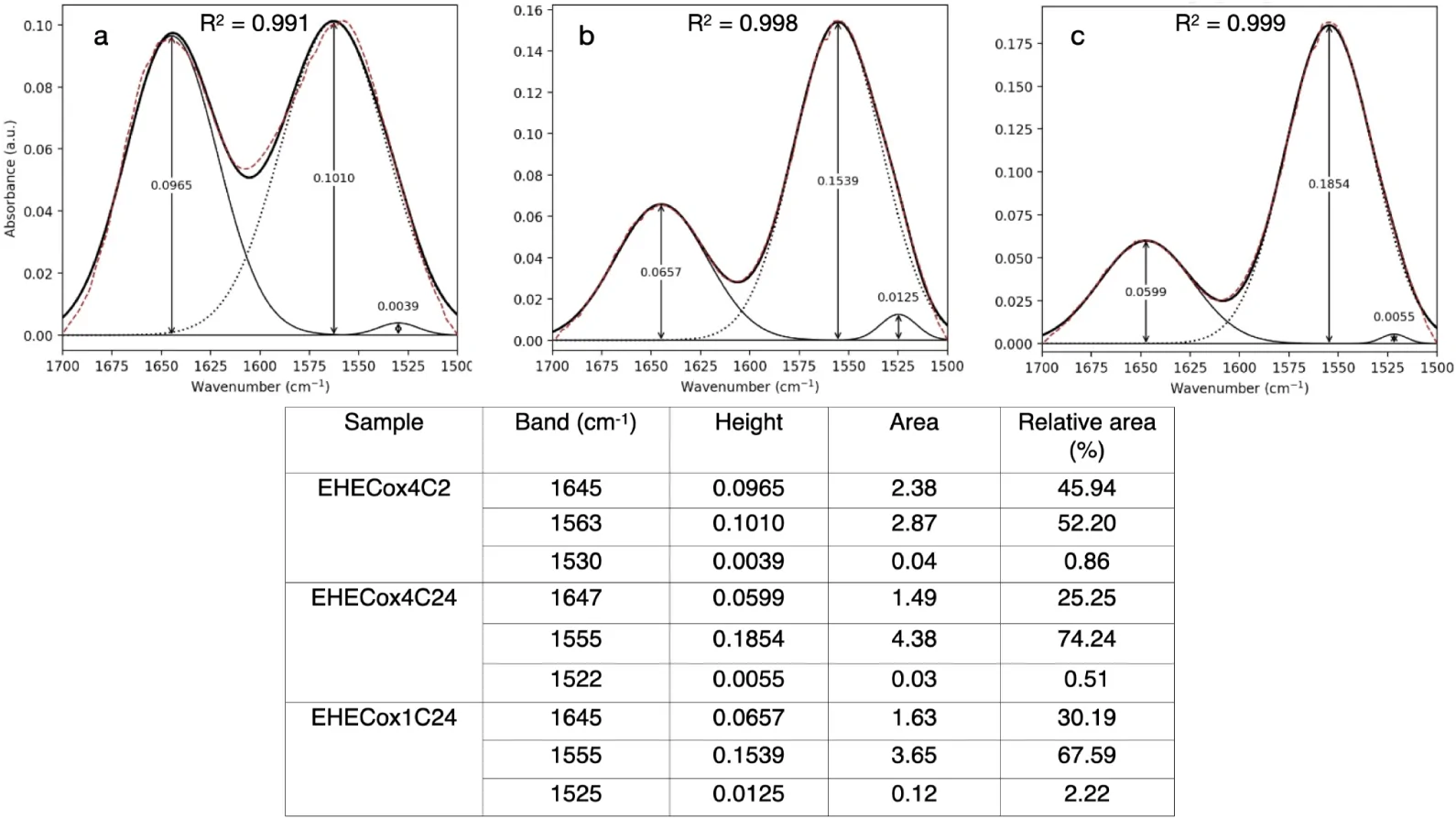
Deconvoluted FTIR of EHEC and chitosan-based films: a) EHECox4C2, b) EHECox1C24 film, and c) EHECox4C24 film.

To evaluate the network densification between EHEC and chitosan, the FTIR spectra within the 1700–1500 cm^–1^ region were deconvoluted into three characteristic sub-bands: Amide I (≈1645 cm^–1^), amide II (≈1555–1562 cm^–1^), and a minor auxiliary band (≈1520–1530 cm^–1^) (Figure 7). The pristine chitosan precursor already shows intrinsic Amide I and Amide II signals due to its residual acetyl groups (degree of deacetylation ∼76%), and quantitative integration of the deconvoluted spectra revealed alterations in this region that depended on the processing parameters. Sample EHECox4C2 exhibited an amide II relative area of 54.20% (total area 2.87). In contrast, extending the UV-cross-linking duration to 24 h (EHECox4C24) significantly increased the absolute area and relative contribution of the amide II band to 74.24% (total area 4.38), suggesting that prolonged UV exposure likely promotes a denser network. Comparing EHECox1C24 with EHECox4C24 shows that a longer oxidation period also influences the network formation (3.65 vs 4.38 for amide II total area). These results suggest that both the degree of prior oxidation and the UV cross-linking duration act synergistically to tailor the reticulation density of the hybrid biomaterial. Although the results are interesting, FTIR alone cannot unambiguously distinguish the possible development of covalent amide linkages from localized changes in hydrogen-bonding microenvironments or polyelectrolyte complexation. Nonetheless, combined with the macroscopic behavior, these spectral trends provide corroborating (albeit indirect) evidence of a densified, cross-linked network.^43,44^

Based on these results, we maintained the EHEC oxidation time at 4 h for investigative and comparative purposes and further investigated samples produced using different cross-linking times.

#### 3.3.3 Further Comparison between EHECox4C2 and EHECox4C24 Samples

The EHECox4C24 sample exhibited an average contact angle of 75.2 ± 0.7°, demonstrating a statistically significant increase (*p* < 0.05) compared to the EHECox4C2 film (62.6 ± 0.5°). This reduction in surface hydrophilicity indicates a denser cross-linked network between the polymer functional groups, yielding a less polar surface. While previous studies on cellulose/chitosan blends report that surface hydrophobicity can be heavily dictated by pH-driven protonation—where increasing acidity alters contact angles due to shifting hydrogen-bonding patterns (e.g., rising from ∼65° to 109° for a 60:40 blend)—our system kept this variable strictly constant for both experiments. Consequently, the observed increase in hydrophobicity is exclusively attributed to the extended UV exposure time (from 2 to 24 h). Furthermore, the contact angle of our 2 h UV-irradiated sample (62.6 ± 0.5°) is remarkably similar to the ∼63° value reported for a comparable cellulose/chitosan mass ratio (∼40:60 at pH 6.3), whereas our 24 h sample achieves a significantly enhanced hydrophobic surface through prolonged UV-induced network densification.^45^

To further investigate structure–property relationships, we evaluated the films’ swelling behavior and water absorption. A key observation was the films’ distinct structural integrity under physical stress. During active handling in an aqueous medium, the EHECox4C2 film maintained its macroscopic integrity for approximately 3 h 45 min, whereas the highly cross-linked EHECox4C24 film remained intact for 6 h 30 min. Under static conditions (without handling), both films remained stable for up to 3 months. Thus, the dense cross-linking in EHECox4C24 significantly enhances the network’s resistance to mechanical disintegration in hydrated environments.

Despite these differences in mechanical resilience, the two samples showed remarkably similar swelling profiles (Figure 8). The EHECox4C2 film (Figure 8a) displayed a multistage water absorption profile: an initial swelling phase, a plateau between 50 and 100 min, and a sharp increase to approximately 1170%. This behavior is characteristic of polymeric hydrogels undergoing solvent diffusion, followed by polymer chain relaxation and the progressive hydration of initially inaccessible domains. The EHECox4C24 sample (Figure 8b) mirrored this absorption profile but exhibited slightly lower swelling values at the final peak. This attenuation aligns with a denser, more cohesive polymer network.^46^

**Figure 8:**
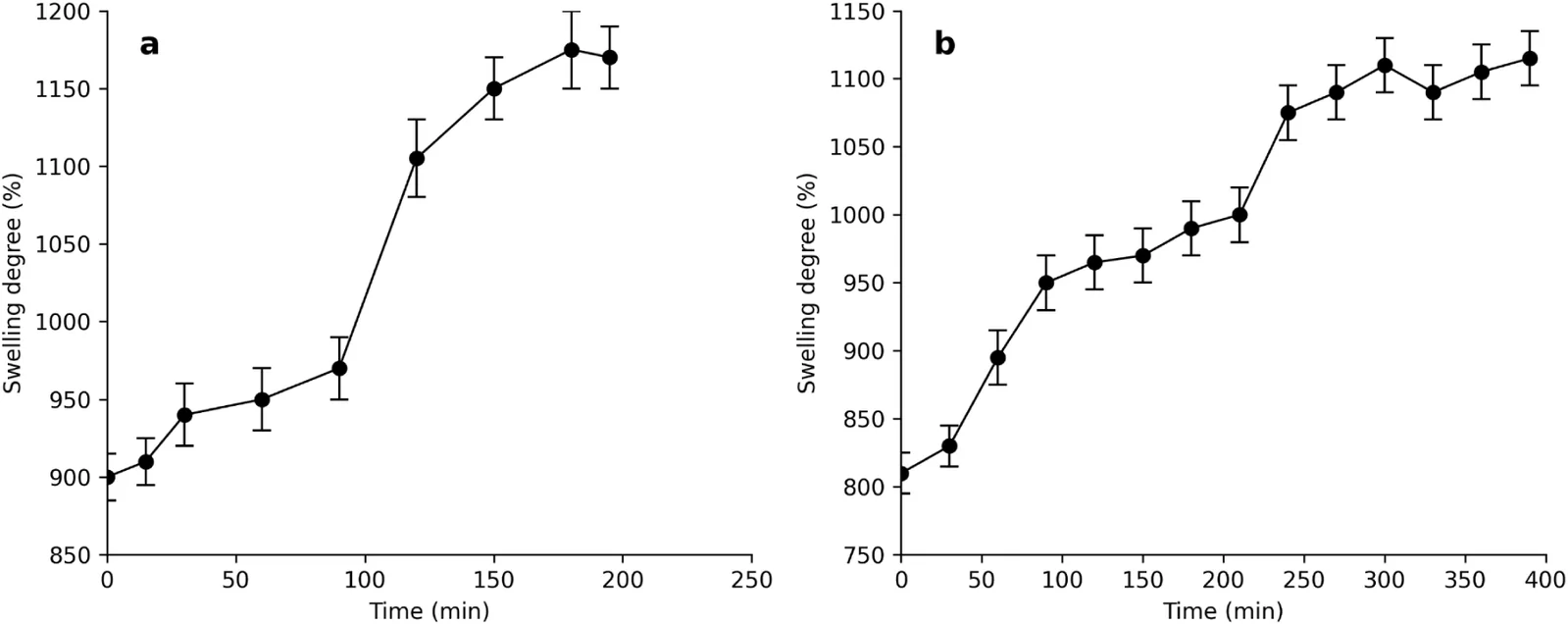
Swelling behavior of the a) EHECox4C2 and b) EHECox4C24 polymer films.

Ultimately, extended EHEC oxidation and prolonged UV exposure increase cross-link density, thereby reinforcing the polymer network. This significantly prolongs structural integrity under mechanical stress while causing only a slight reduction in overall swelling. This demonstrates that tuning synthesis parameters effectively optimizes the material’s stability without compromising its fundamental architecture or fluid absorption capacity. Consequently, these robust formulations seem to be particularly suitable for demanding aqueous applications, such as tissue engineering and controlled-release matrices, where long-term durability outweighs the need for maximum fluid absorption.^47,48^

The thermal stability of the hybrid films was evaluated by thermogravimetric analysis (TGA) and derivative thermogravimetry (DTG) and compared to that of pure EHEC and chitosan (Figure 9).

**Figure 9:**
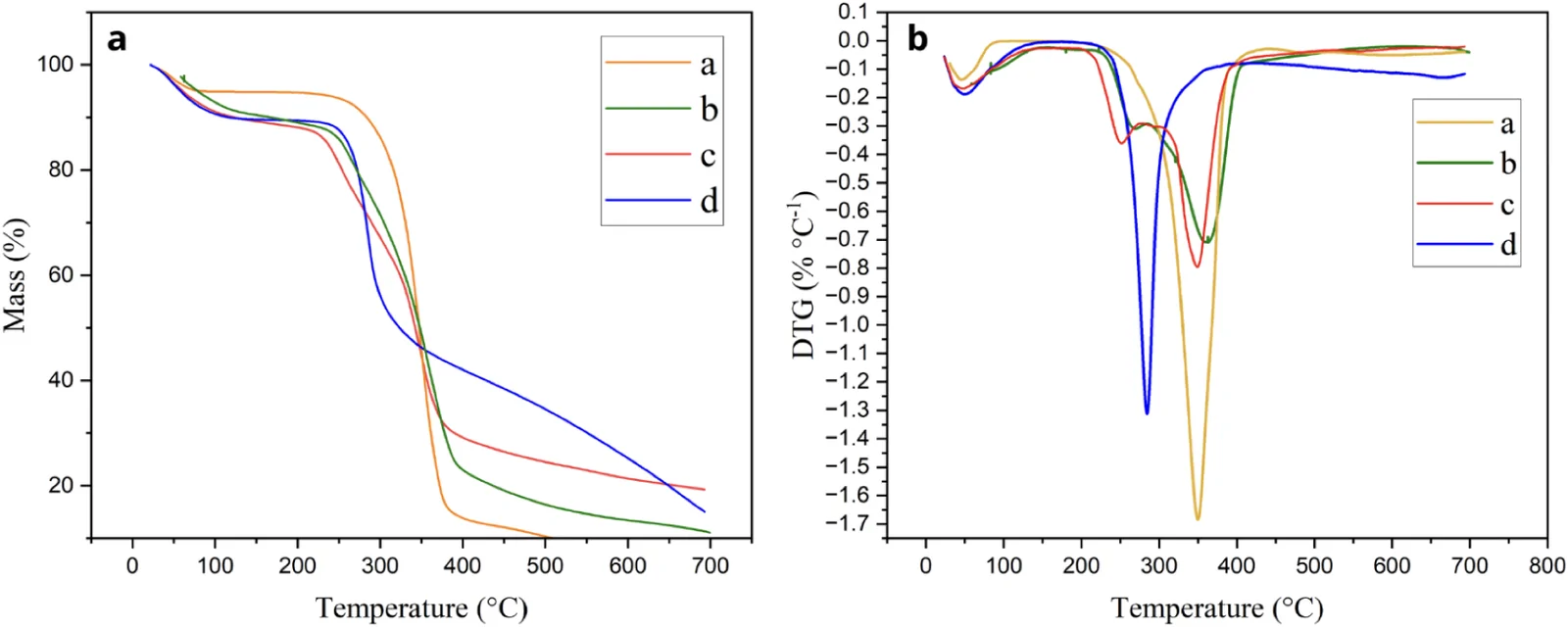
Thermogravimetric (TGA) and derivative thermogravimetric (DTG) analyses of the samples: EHEC, EHECox4C2, EHECox4C24, and chitosan, respectively. Panel (a) shows the TGA curves, and panel (b) shows the corresponding DTG curves.

The TGA and DTG analyses show clear differences in the thermal behavior of the precursors and the hybrid films. All samples exhibit an initial mass-loss stage below 120 °C, associated with the removal of physically adsorbed water, followed by the main degradation stage between 250 and 380 °C, attributed to the degradation of the main chains of both polymers (Figure 9a). The maximum degradation-rate temperature (*T*_max_) (Figure 9b) for pure chitosan was 284.7 °C, showing the lowest thermal stability, while pure EHEC exhibited a *T*_max_ of 350.68 °C. Regarding the hybrid films, EHECox4C2 exhibited a maximum degradation temperature of 362.82 °C, while the EHECox4C24 film showed a *T*_max_ of 350.47 °C, which closely resembles the peak temperature of pure EHEC. However, although EHECox4C2 exhibited the highest *T*_max_, the TGA curve of the EHECox4C24 film shows a shift in the thermal degradation onset temperature toward higher values, indicating enhanced initial thermal resistance. Furthermore, EHECox4C24 decomposes more gradually, with a lower mass-loss rate during the main degradation step, suggesting the formation of a denser, more stable polymer network. At high temperatures, EHECox4C24 also yields a higher carbonaceous residue than EHECox4C2.^49,50^

Regarding thermal stability, both systems present significant advantages over other cross-linked polysaccharide blends. Previous studies on chitosan/HEC films chemically cross-linked with epichlorohydrin, for example, have reported a notable decrease in thermal stability upon cross-linking, with maximum decomposition temperatures (*T*_max_) of approximately 183 °C.^51–53^

The EHECox4C24 film was chosen to expand the study because of its superior homogeneity. While 24 h of irradiation represents the optimal condition when matrix uniformity and wet durability are paramount, the 2 h treated film serves as an energy-saving alternative for passive water barrier applications.

### 3.4 Further Characterization of EHECox4C24 Films

#### 3.4.1 Submersion in Different Media

We monitored the mass loss of the EHECox4C24 film over an 18-day submersion period in deionized water. We observed a rapid mass reduction of 67.9% ± 12.5% within the first 24 h. Subsequently, we observed stabilization of mass loss, recording values of 60.1% ± 10.3% at 5 days, 63.8% ± 1.9% at 7 days, and 59.2% ± 10.1% at 18 days. Considering the standard deviations and the presence of hydration during this period, these results confirm a biphasic behavior characterized by an initial burst solubilization—likely corresponding to the leaching of noncovalently cross-linked polymer chains—followed by a highly stable phase.

As the submersion period extended to 30 days, the film exhibited pronounced swelling, absorbing water to the point of behaving like a highly hydrated gel. This superabsorbent state compromised its physical rigidity, making it impossible to reliably remove, dry, and weigh the sample for further gravimetric measurements without destroying the network. Nevertheless, despite the initial leaching of the soluble fraction and the extensive subsequent swelling, the core cross-linked network remained stable. The resulting material demonstrated a controlled solubility profile, retaining its macroscopic structural integrity in an aqueous medium for up to 3 months.

The films obtained were also subjected to solubility tests in aqueous solutions at pH 2–14 to evaluate the effects of acidic, neutral, and basic media on the material’s integrity and resistance. In solutions adjusted to pH 2 and 3 with acetic acid, the EHECox4C24 film was completely solubilized within 30 min. However, the film remained stable at pH 4, with only loss of the characteristic yellowish coloration. At pH 6 (unadjusted) and at pH 5, 8, 10, 11, 12, 13, and 14 (adjusted with NaOH), the films remained structurally intact and retained their yellowish color. These results indicate that film solubility occurs only under highly acidic conditions (pH < 3), consistent with amide bond hydrolysis and the protonation of chitosan amine groups, which also affects intermolecular interactions.^54^ Furthermore, we observed that films maintained at pH levels above 10 showed greater structural integrity over time than in previously performed tests, which maintained their integrity for up to 5 months (Supporting Information, Figure S4). This behavior suggests that cross-linking between EHECox and chitosan occurred primarily through stable amide formation, conferring resistance to solubilization in neutral and basic environments and sensitivity to degradation only under extreme acidity.

An additional solubility test was performed in organic solvents using chloroform, acetonitrile, and dimethyl sulfoxide (DMSO). The film remained insoluble under all conditions tested. The insolubility of the EHECox4C24 film in DMSO—a solvent known to disrupt strong intermolecular hydrogen bonds in polysaccharides—indirectly suggests that the network’s integrity transcends secondary physical interactions. Simultaneous insolubility in a nonpolar solvent (chloroform), a moderately polar aprotic solvent (acetonitrile), and a strongly polar aprotic solvent (DMSO) indicates a high degree of cross-linking in the polymer matrix. In particular, the insolubility in DMSO, a solvent with a high dielectric constant and a strong ability to form hydrogen bonds, suggests that the network has robust structural links, such as covalent cross-links and/or a dense hydrogen-bond network.^55^

#### 3.4.2 XRD Analyses

The X-ray diffractogram of purified EHEC (Figure 9a) displays a broad, diffuse halo devoid of well-defined Bragg peaks, a hallmark of predominantly amorphous materials. This profile indicates an absence of long-range structural order and evident crystalline domains. Such a pattern is widely reported for cellulose derivatives, arising from hydrogen-bond rupture and partial destruction of crystalline regions during chemical modification and purification.^56^ A comparable amorphous profile is observed for the EHECox4C24 film (Figure 10b). This demonstrates that oxidation and subsequent chemical cross-linking did not induce a reorganization of the polymer chains sufficient to reestablish crystallinity. Rather, the introduction of oxygenated functional groups disrupts the regularity and packing of the cellulose chains, further favoring an amorphous state.^56,57^

**Figure 10:**
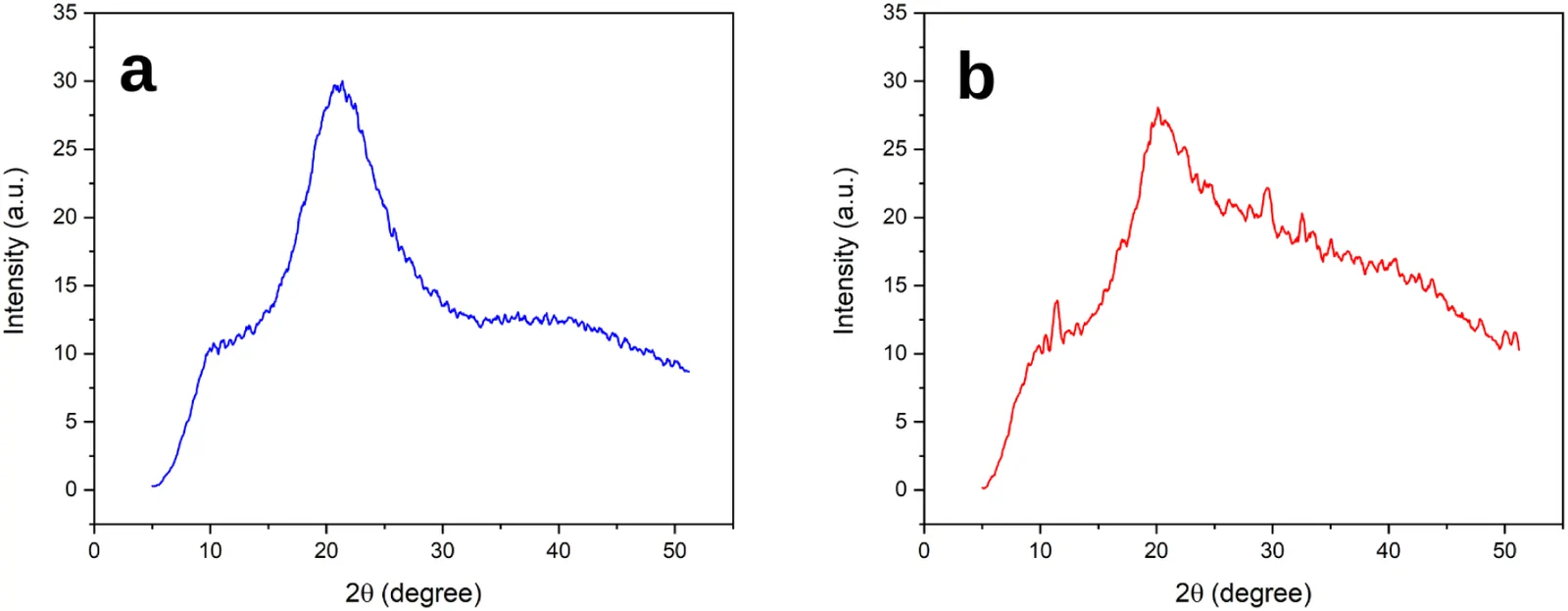
X-ray diffractograms of a) EHEC films and b) EHECox4C24.

Therefore, both the purified EHEC and the EHECox4C24 films exhibit essentially amorphous structural characteristics. This is consistent with expectations for modified cellulose derivatives, in which chemical functionalization disrupts the supramolecular network originally present in native cellulose.^58^

#### 3.4.3 SEM Analysis

In the micrograph obtained at 1000× magnification (Figure 11a), a predominantly homogeneous surface is observed, with no evident fissures and good matrix continuity, indicating that the film-forming process was, for the most part, efficient. Despite this overall uniformity, small, dispersed particles are observed, possibly related to residues from the preparation process, as reported in other studies that used acetic acid to solubilize chitosan. At 2500× magnification (Figure 11b), the surface reveals additional details, such as local roughness and agglomeration points, which stand out against the continuous matrix. A plausible interpretation is that some of these particles correspond to acetate salts, derived from the acetic acid used to solubilize chitosan, as reported in studies on the influence of different acids on film morphology.^59,60^ If there is no complete evaporation or neutralization, these residues may remain incorporated into the film’s final structure, altering its properties.

**Figure 11:**
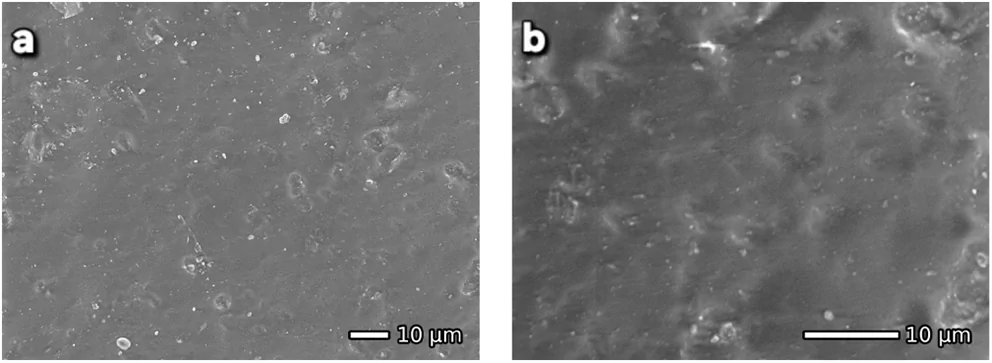
SEM of the surface of the EHECox4C24 film, a) 1000× magnification; b) 2500× magnification.

Overall, the results indicate that the films exhibit good homogeneity and structural cohesion, desirable characteristics for polymeric material applications. However, microdefects and surface residues can act as weak points, accounting for the lower tensile strength and elongation observed in mechanical tests compared with those reported for pure chitosan films in the literature.^61^

#### 3.4.4 Mechanical Properties

Tensile tests on the chitosan-cross-linked EHECox specimens (thickness: 0.07–0.10 mm, gauge length: ∼45–48 mm) revealed predominantly rigid, brittle behavior. The stress–strain curves (Supporting Information, Figure S5) show limited deformation followed by abrupt fracture, resulting in an average elongation at break of 7.2 ± 1.5%. This brittleness is commonly observed in unplasticized, high-cellulose systems where dense hydrogen-bond networks and UV-induced cross-linking severely restrict polymer chain mobility.^62,63^ Furthermore, structural heterogeneities and microcracks potentially introduced during the bio-oxidation process may compromise overall integrity.^10^ It is also worth noting that the applied strain rate can influence these viscoelastic materials, potentially underestimating ductility if the rate does not match the polymer’s relaxation time.^64^

For comparative purposes, films of purified EHEC and pure chitosan were evaluated under identical conditions (Supporting Information, Figures S6 and S7). Visually, the purified EHEC appeared translucent and flexible, whereas pure chitosan was slightly more opaque, homogeneous, and tear-resistant during handling.

As detailed in Table 2, purified EHEC exhibited the lowest mechanical performance, indicating a less cohesive matrix. Conversely, pure chitosan displayed the highest tensile strength and a slight increase in deformation capacity. The cross-linked EHECox4C24 film bridges the gap between these precursors. The UV-induced cross-linking with chitosan promoted greater structural cohesion and efficient load transfer between chains, significantly increasing the elastic modulus (*p* < 0.05) compared to pure EHEC. The stiffness of the cross-linked film reached levels statistically comparable to pure chitosan (both labeled with the superscript “a” in Table 2). Although the cross-linked film showed a numerical increase in tensile strength and elongation, statistical analysis showed these differences were not significant (*p* > 0.05) because of the data dispersion inherent to the brittle nature of these highly cross-linked polysaccharide networks.^51,65^ Rather than a general mechanical improvement, the EHECox4C24 system enhances stiffness and strength at the expense of flexibility. This trade-off is common in biopolymer composites, as simultaneously increasing stiffness and flexibility remains a significant challenge in the literature. For instance, recent studies employing double-network hydrogels of chitosan and hydroxyethyl cellulose (HEC) cross-linked with citric acid and cinnamaldehyde reported highly flexible films (elongation at break of 45.02%) but lower mechanical resistance, with tensile strengths around 9.52 MPa.^66^ On the other end of the mechanical spectrum, Chitosan/HEC composite films cross-linked with epichlorohydrin (ECH) and loaded with CuO nanoparticles have been reported to reach very high tensile strengths (up to 77.02 MPa). However, this intense cross-linking severely restricts chain mobility and causes brittleness, with elongations dropping to 3.0–3.5%. Furthermore, the reliance on ECH poses significant environmental and biological toxicity concerns, limiting biomedical applications.^51^

**Table 2:** Mechanical Properties: Elastic Modulus, Elongation at Break, and Tensile Strength[Table-fn tbl2fn1]

Sample	Elastic modulus (MPa)	Elongation at break (%)	Tensile strength (MPa)
EHECox4C24	775.6 ± 172.8^a^	7.2 ± 1.5^a^	21.6 ± 4.6^a^
Chitosan	752.9 ± 118.6^a^	8.2 ± 2.2^a^	23.7 ± 9.2^a^
EHEC	537.8 ± 114.6^b^	6.1 ± 1.1^a^	14.7 ± 5.6^a^

i Values represent mean ± standard deviation (*n* = 5). Different lowercase letters in the same column indicate statistically significant differences (*p* < 0.05).

Ultimately, chitosan cross-linking reinforces the EHEC matrix, whether through new hydrogen bonds or possible covalent bond formation, significantly increasing rigidity without compromising the material’s fundamental mechanical integrity and maintaining the typical unplasticized biopolymer profile. The EHECox4C24 system, however, achieves this result without cross-linking agents, offering a green alternative to the more complex or hazardous chemical modifications previously reported for cellulose and chitosan derivatives.^18^

#### 3.4.5 Cytotoxicity Assay

To evaluate the cytotoxicity of the obtained films, the material was biologically characterized using cytotoxicity assays by extract and direct contact with L929 fibroblast cells, according to ISO 10993-5 guidelines. The assays were conducted with both washed and unwashed samples to investigate the influence of residues from the film-production process on cell viability.

In the cytotoxicity evaluation of the extract (Figure 12a), the unwashed material showed reduced cell viability at the 50% and 100% concentrations, with values below the 70% limit established by ISO 10993-5. These results indicate residual compounds released during the film’s initial hydration. However, after washing the material with PBS for 2 h, none of the tested concentrations showed reduced metabolic activity relative to the control, indicating that a simple wash efficiently removes cytotoxic leachables.

**Figure 12:**
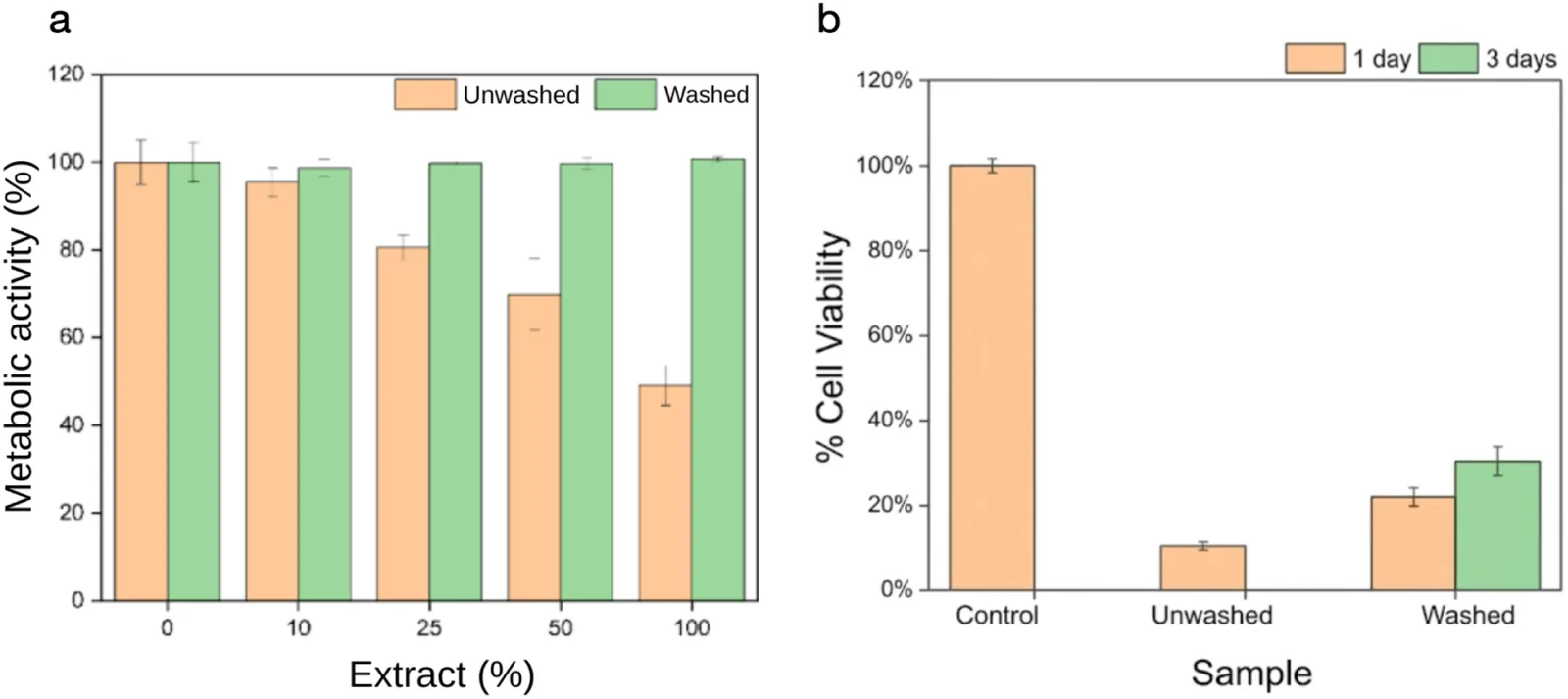
(a) Evaluation of MTS cytotoxicity of the extract in L929 fibroblasts after 24 h of treatment. The negative control (−) represents 100% cell viability. (b) Cytotoxicity evaluation using an MTS reduction assay in L929 fibroblasts cultured on the material for 1 and 3 days. The control corresponds to the culture plate after 24 h of adhesion.

The cytotoxicity observed in unwashed chitosan films may be due to residual acetic acid from the chitosan solubilization process. Studies show that chitosan films prepared with various organic acids, including acetic acid, can reduce fibroblast cell viability at high concentrations, suggesting that acid residues directly influence cytotoxicity.^67^ Acetic acid solutions, even at low concentrations, can cause cytotoxic effects in cells, reinforcing the role of this component in the observed decrease in viability.^68,69^

In the direct contact assay (Figure 12b), the unwashed material showed severely reduced cellular metabolic activity after 24 h of contact, suggesting poor adhesion and interference from toxic components on the film surface. Although the washed material showed a slight improvement and a progressive increase in metabolic activity between 24 and 72 h compared with the unwashed samples, overall cell viability remained considerably lower than that of the control.

These findings distinguish extract-mediated responses from direct cell-surface interactions. After washing, EHECox4C24 film extracts supported high cellular metabolic activity (∼100%). The lower viability observed in the direct-contact assay (∼20–30%) likely reflects poor initial adhesion and spreading of anchorage-dependent L929 cells on the hydrated and hydrophilic matrix, which does not necessarily indicate intrinsic chemical cytotoxicity of the material itself. While this characteristic limits the film’s use as a cell-anchoring scaffold in its current form, it suggests the material could be explored for nonadherent interfaces.

#### 3.4.6 Biodegradability in Soil

The preliminary susceptibility of the EHECox4C24 film to environmental breakdown was evaluated through a simulated soil burial test over 4 months. After incubation, the films became visibly thinner and developed a rough, irregular surface (Figure 13), indicating surface degradation and physical interaction with the support mesh.

**Figure 13:**
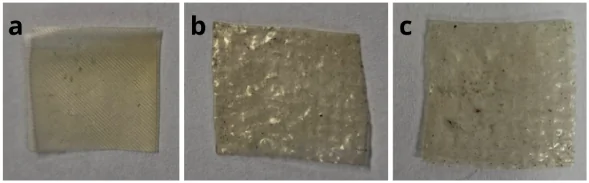
Sample of the EHECox4C24 films: a) before, b) after 2 months, and c) after 4 months of biodegradability testing in soil.

After 2 months of incubation (Figure 12b), the films showed a mass loss of 6.21 ± 0.51%, likely reflecting preferential cleavage of susceptible amorphous regions.^70,71^ By the fourth month (Figure 13c), mass loss increased to 8.47 ± 0.43%, demonstrating continued but decelerated biodegradation. This reduced degradation rate was likely limited by water availability rather than pH fluctuations. Irrigation was restricted to once a week, which may have been insufficient to maintain optimal soil moisture for microbial activity, particularly during hotter periods. Conversely, the soil pH remained stable at approximately 6.0, a slightly acidic condition that generally favors microbial activity and organic matter decomposition, suggesting that moisture was the primary limiting factor.^71^

FTIR analysis (Figure 14) corroborated the macroscopic observations by revealing structural modifications in the polymer network.

**Figure 14:**
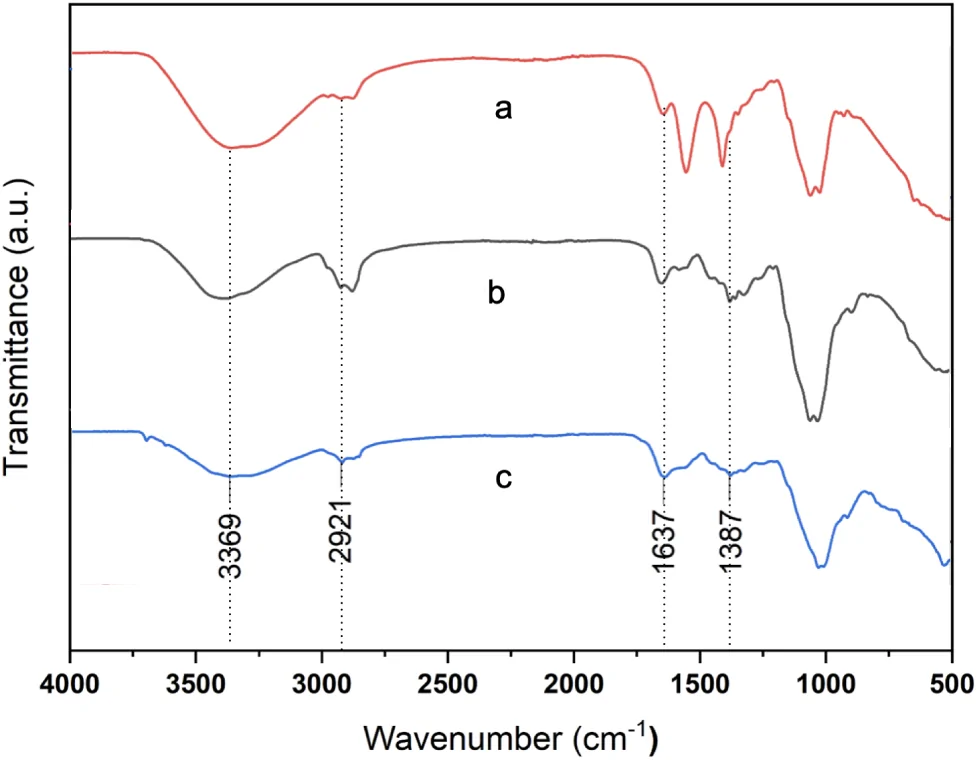
EHECox4C24 film a) before submersion in soil, b) after 2 months of biodegradation in soil, and c) after 4 months of biodegradation in soil.

After biodegradation, the reduction of the broad band at 3410–3420 cm^–1^ (O–H and N–H stretching) indicated fewer free hydroxyl groups, possibly due to oxidation. Concurrently, the diminished intensity of the carbonyl band (∼1640 cm^–1^) suggested the transformation of C=O groups, potentially leading to CO_2_. Furthermore, subtle shifts in the 1200–1000 cm^–1^ region (C–O–C vibrations) were observed and may relate to cleavage of glycosidic bonds during polysaccharide backbone breakdown. The postincubation spectra also exhibited peaks overlapping the 3700–3200 cm^–1^ region, along with distinct vibrations between 1200 and 900 cm^–1^. These changes are frequently associated in the literature with the physical integration of earthy particles onto the partially degraded film during the degradation process.^72–75^

Despite the promising properties of the EHECox4C24 films, future studies must acknowledge the current system’s limitations. The dense cross-linked network inherently contributes to the material’s brittleness (elongation at break of ∼7%), which restricts its use in packaging applications that demand high flexibility; further tests with plasticizers would be interesting. Environmentally, the biodegradation rate in soil was relatively slow (∼8.5% mass loss over four months), indicating that although the material is biodegradable, industrial composting conditions with higher moisture and microbial load might be necessary for rapid degradation. Furthermore, the film’s structural integrity is compromised in highly acidic media (pH < 3), limiting its use in highly acidic environments. Although the material shows no soluble cytotoxic leachables, its low direct cell attachment makes it a nonadherent material in its current form. If cellular infiltration and tissue scaffold integration are desired, surface functionalization would be mandatory. Finally, from a manufacturing perspective, scaling up UV-based processes requires strict turbidity control and regular lamp cleaning to prevent drastic efficiency drops. Future optimizations, such as combining UV sources with green photoinitiators, could significantly reduce curing times for large-scale applications.

## 4 Conclusions

This study demonstrated that cross-linking EHEC with chitosan after enzymatic bio-oxidation is an effective strategy for producing polymeric films with improved structural, thermal, and hydrolytic properties.

Laccase/TEMPO-mediated oxidation of EHEC introduced reactive carboxyl functional groups, enabling the formation of a robust, aqueous-stable polymeric network with chitosan, likely through covalent cross-linking and/or synergistic supramolecular interactions. Reaction times of 1 to 4 h yielded predominantly similar results.

Among the methodologies evaluated for EHECox cross-linking with chitosan, UV light cross-linking proved most efficient, yielding films with greater structural integrity in aqueous media and greater resistance to solubilization than the other methods tested. The formulation containing 50% w/w chitosan relative to EHECox performed best, showing that composition strongly influences the stability, mobility, and cohesion of the polymeric matrix. Regarding cross-linking time, 24 h was identified as optimal when prioritizing maximum wet-handling stability and structural homogeneity, without compromising the high fluid-swelling capacity (∼1100%). However, this parameter remains flexible and can be shortened depending on the specific requirements of the target application. The 2 h irradiation condition remains a viable, energy-efficient alternative for applications where passive aqueous stability is sufficient, but processing throughput is critical

Films produced by the optimized process showed high resistance to solvation in various organic solvents and stability over a wide pH range in aqueous media, maintaining integrity for extended periods, especially in alkaline media (3 to 5 months, depending on pH). Thermal analyses confirmed adequate stability for moderate-temperature applications; mechanical tests demonstrated an enhancement in elastic modulus (stiffness) relative to pure EHEC, although the material remains brittle (elongation ∼7%) due to restricted chain mobility inherent to the unplasticized cross-linked network; and biological assays showed no release of soluble cytotoxic leachable compounds into the surrounding medium, allowing future investigations and modifications for biomedical applications. The predominantly amorphous structure observed by XRD was consistent with the system’s chemical functionalization and the partial biodegradation observed in soil.

Overall, the results confirm that combining EHECox and chitosan, cross-linked under UV light irradiation, represents a promising, environmentally friendly, and technically feasible approach to developing biomaterials with aqueous and thermal stability. Future studies could explore incorporating bioactive agents, using nanometric reinforcements, and evaluating these materials in simulated physiological media to enhance performance and broaden their applications.

## Supplementary Material



## Notes

Photograph of the EHECox4C24 sample submerged in water for 3 months (Figure S1); conductometric titration curves of films obtained after EHEC oxidation (1–4 h) and cross-linking with chitosan under UV light irradiation for 2 h (Figure S2); visual aspect of EHECox films containing 25% and 50% chitosan (Figure S3); photograph of EHECox4C24 submerged in an alkaline solution (pH 10) for 5 months (Figure S4); appearance of EHECox4C24 films after mechanical rupture (Figure S5); comparison of purified EHEC and pure chitosan films (Figure S6); and stress vs strain curves for chitosan, EHECox4C24, and purified EHEC (Figure S7) (PDF)
