# Supplementary material for: Enzymatic Bio-Oxidation of Ethyl Hydroxyethyl Cellulose and UV-Assisted Cross-Linking with Chitosan for Enhanced Aqueous Stability in Biomaterial Production
Source: ACS Omega. 2026 Oct 19;11(43):67433–47. doi: 10.1021/acsomega.6c08601 (PMC13736844; doi:10.1021/acsomega.6c08601)
Supplement: Supplementary file 1 [file ao6c08601_si_001.pdf]

Enzymatic biooxidation of ethyl hydroxyethyl cellulose and UV-assisted crosslinking with chitosan for enhanced aqueous stability biomaterial production.

*Beatriz Fernanda da Silva,[a] Lidiane Meier,[a] Ismael Casagrande Bellettini,[a] Karina Cesca,[b] Ana Paula Serafini Immich,[b] Patrícia Bulegon Brondani[c]**

[a] Departamento de Ciências Exatas e Educação, Centro Tecnológico, de Ciências Exatas e Educação, Universidade Federal de Santa Catarina, Rua Marechal Rondon, 880 - Salto do Norte, Blumenau, 89065-200, Blumenau, SC, Brasil

[b] Departamento de Engenharia Química e Engenharia de Alimentos, Centro Tecnológico, Córrego Grande, Florianópolis, 88040-900, SC, Brasil

[c]Departamento de Química, Centro de Ciências Físicas e Matemáticas,Universidade Federal de Santa Catarina, Rua Roberto Sampaio Gonzaga, s/n - Trindade, Florianópolis, 88040-380, SC, Brasil

Supporting Information

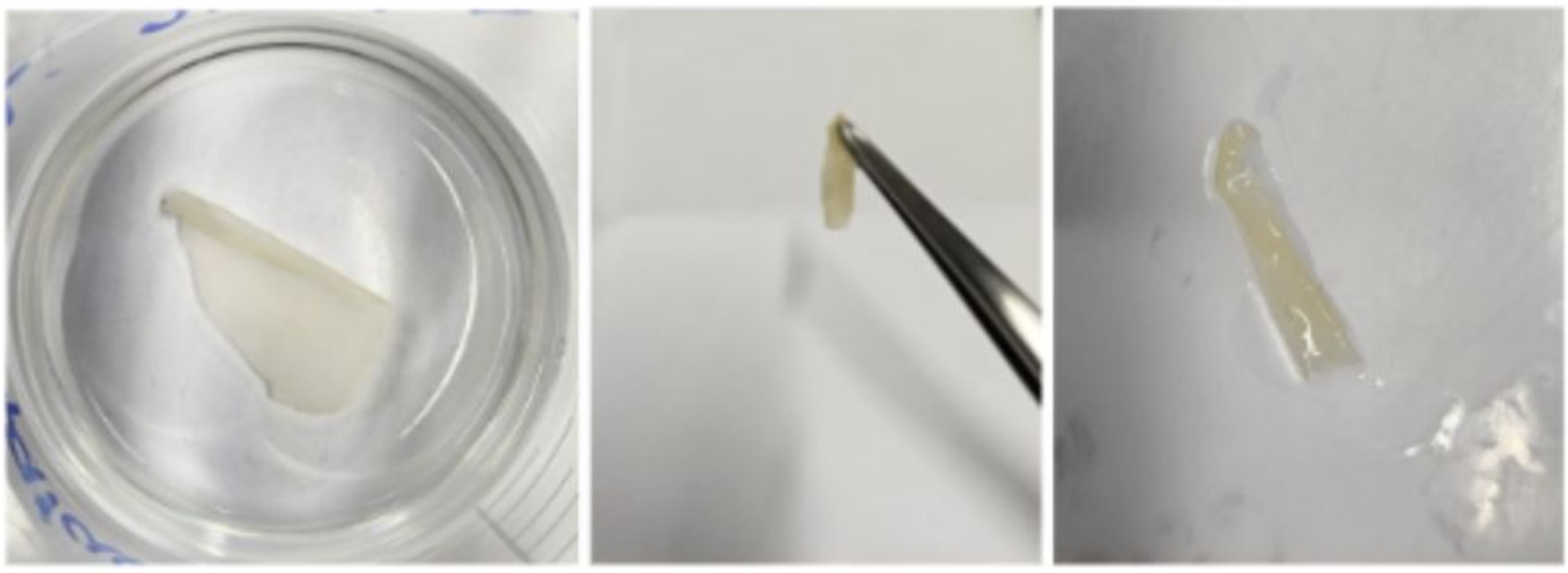

Figure S1 – Picture of sample of EHECox4C24 submerged in water for three months.

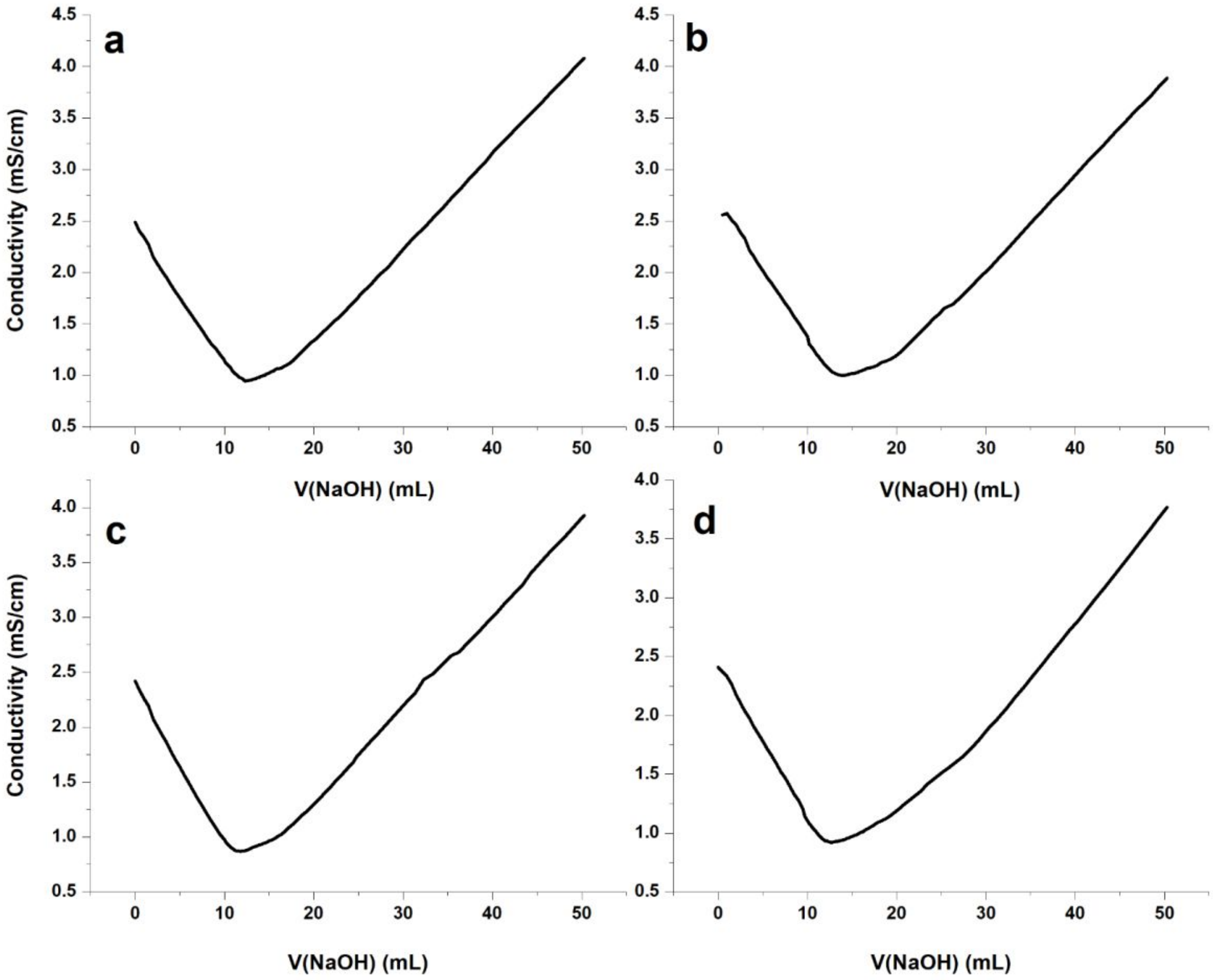

Figure S2. Graphs of the conductometric titration of films obtained after EHEC oxidation for 1-4 h and cross-linking with chitosan under UV light irradiation for 2 h. a) 1 h; b) 2 h; c) 3 h; d) 4 h.

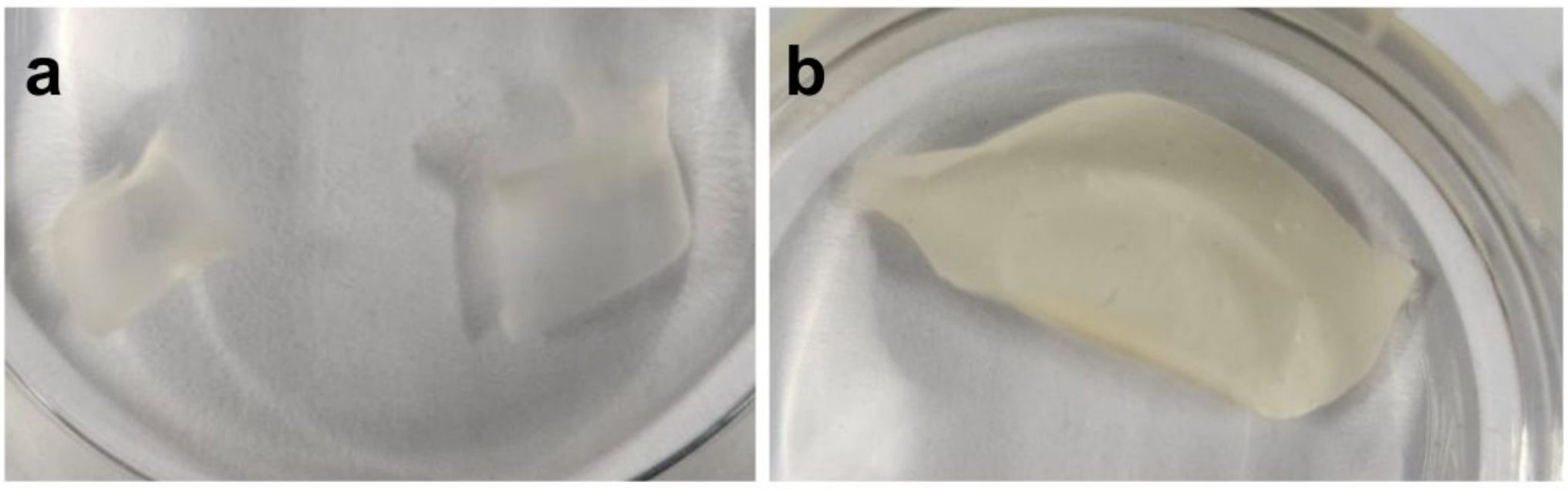

Figure S3. a) EHECox film with 25% chitosan, and b) EHECox film with 50% chitosan.

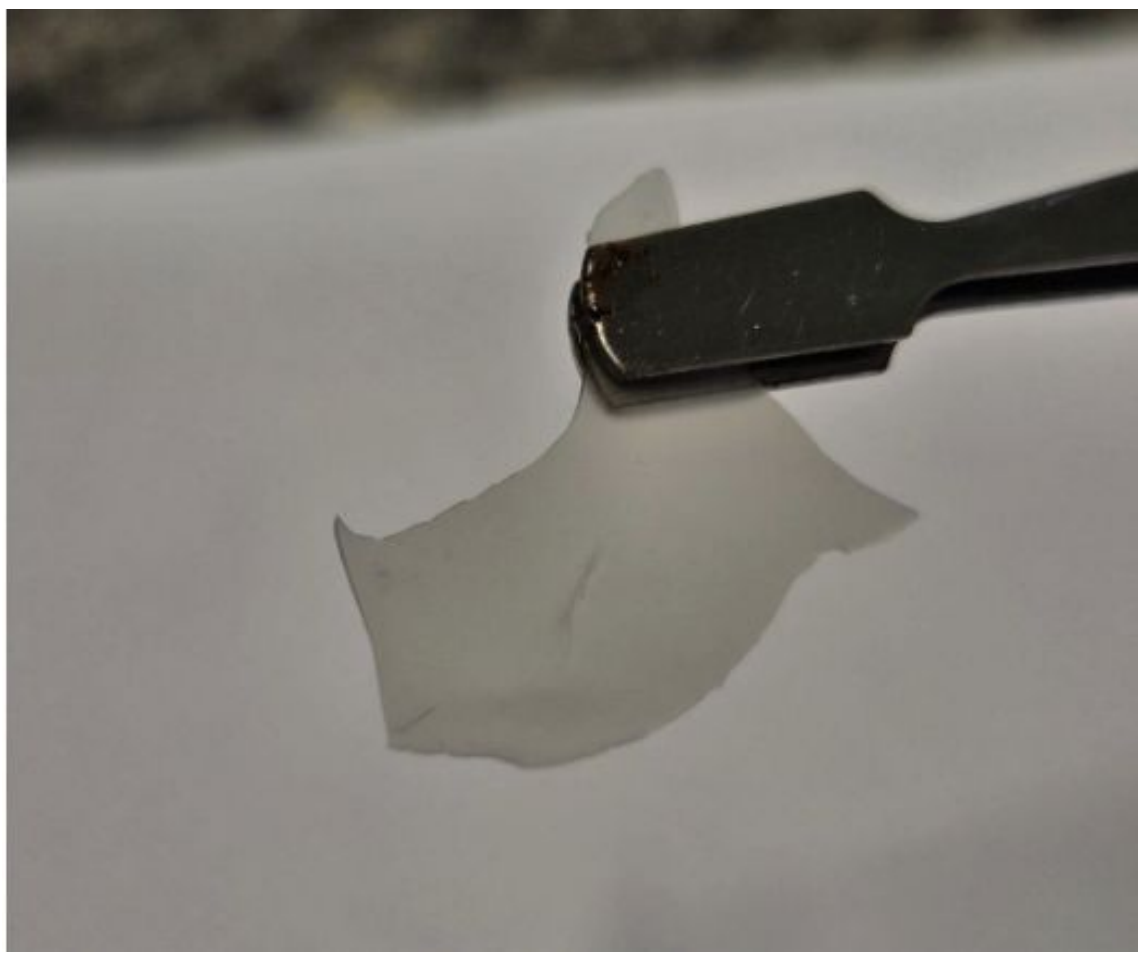

Figure S4 - EHECox4C24 in an alkaline solution (pH 10) submerged for five months.

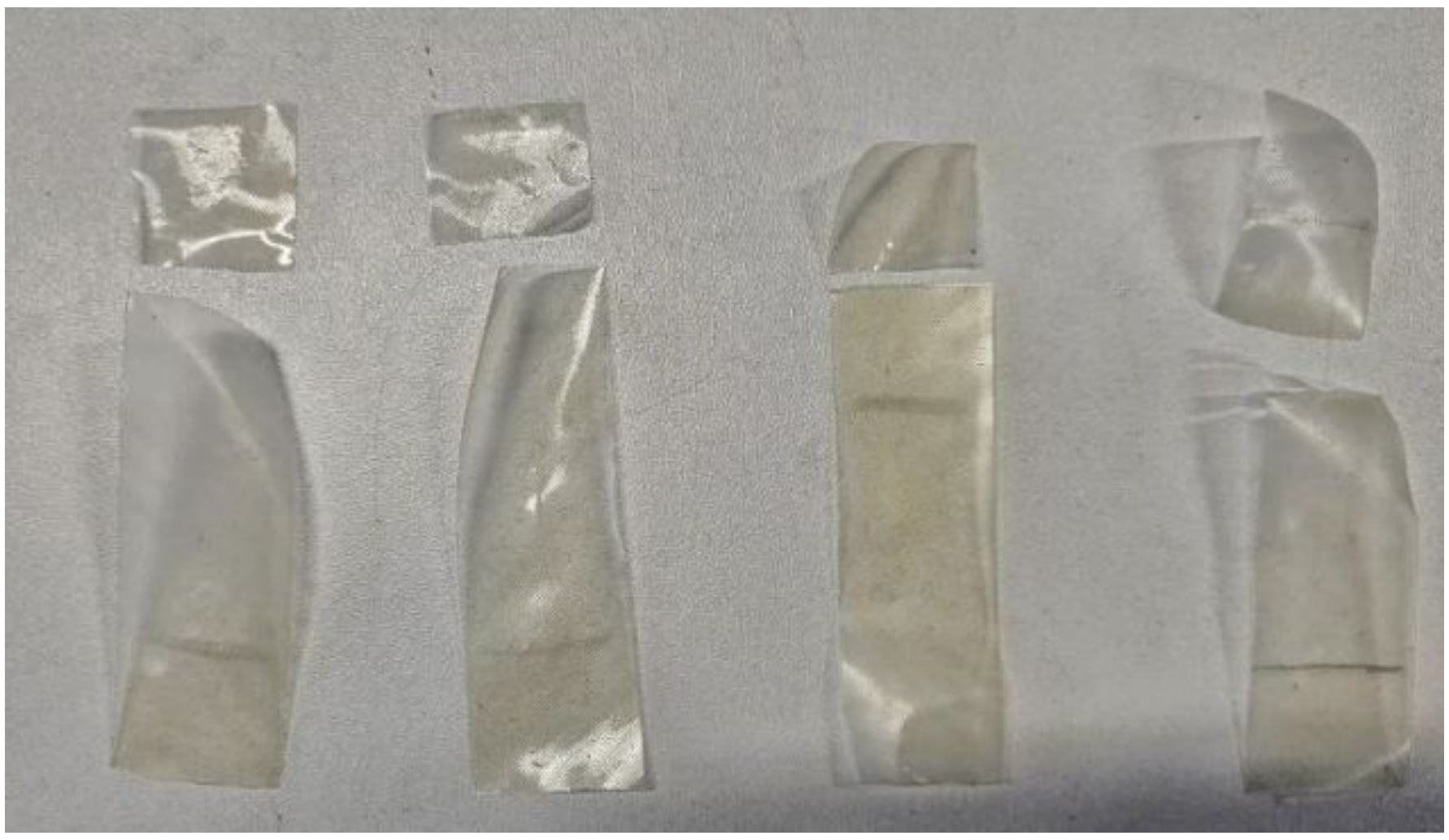

Figure S5. EHECox4C24 films after rupture

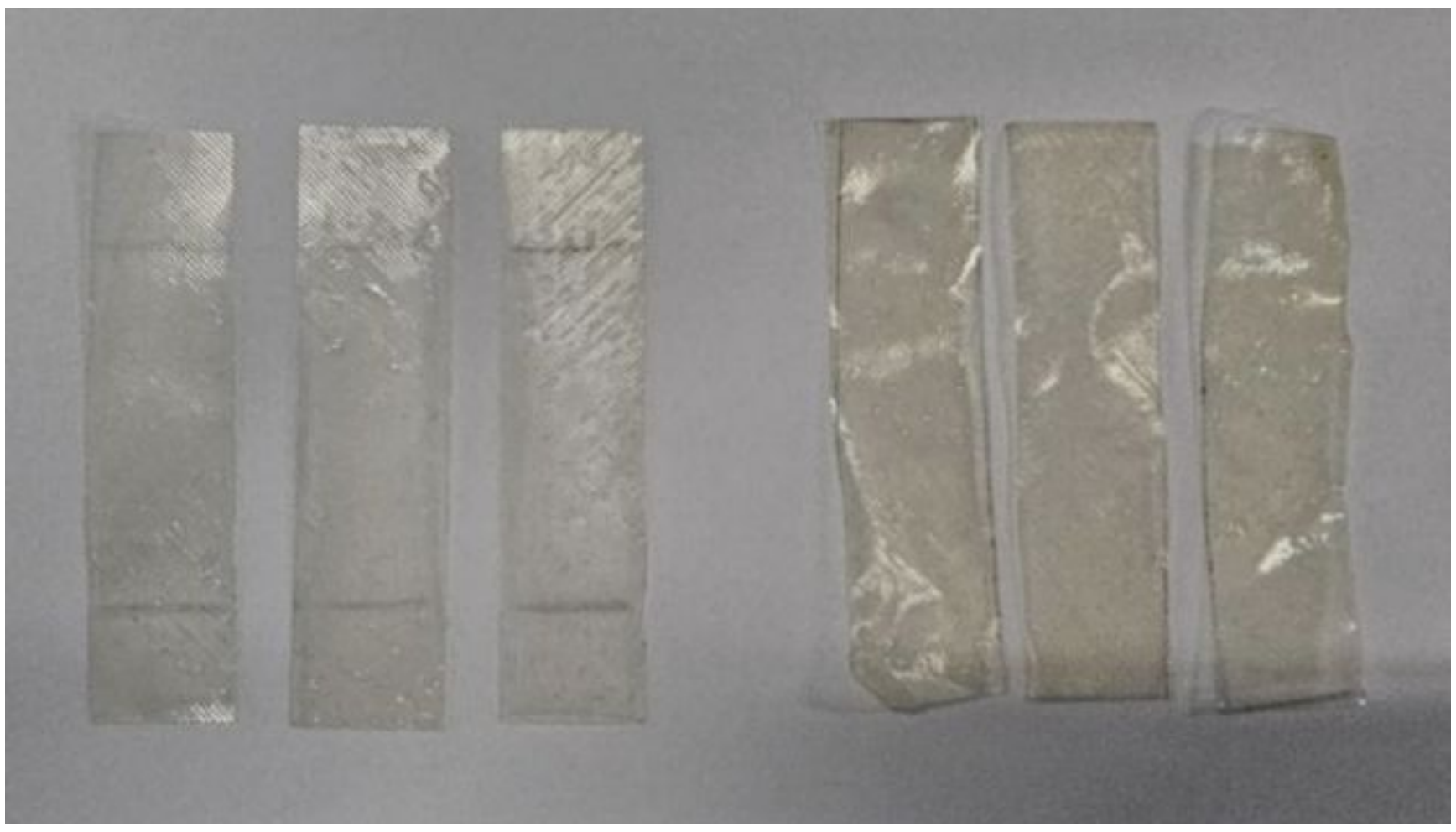

Figure S6. Purified EHEC films on the left and pure chitosan films on the right.

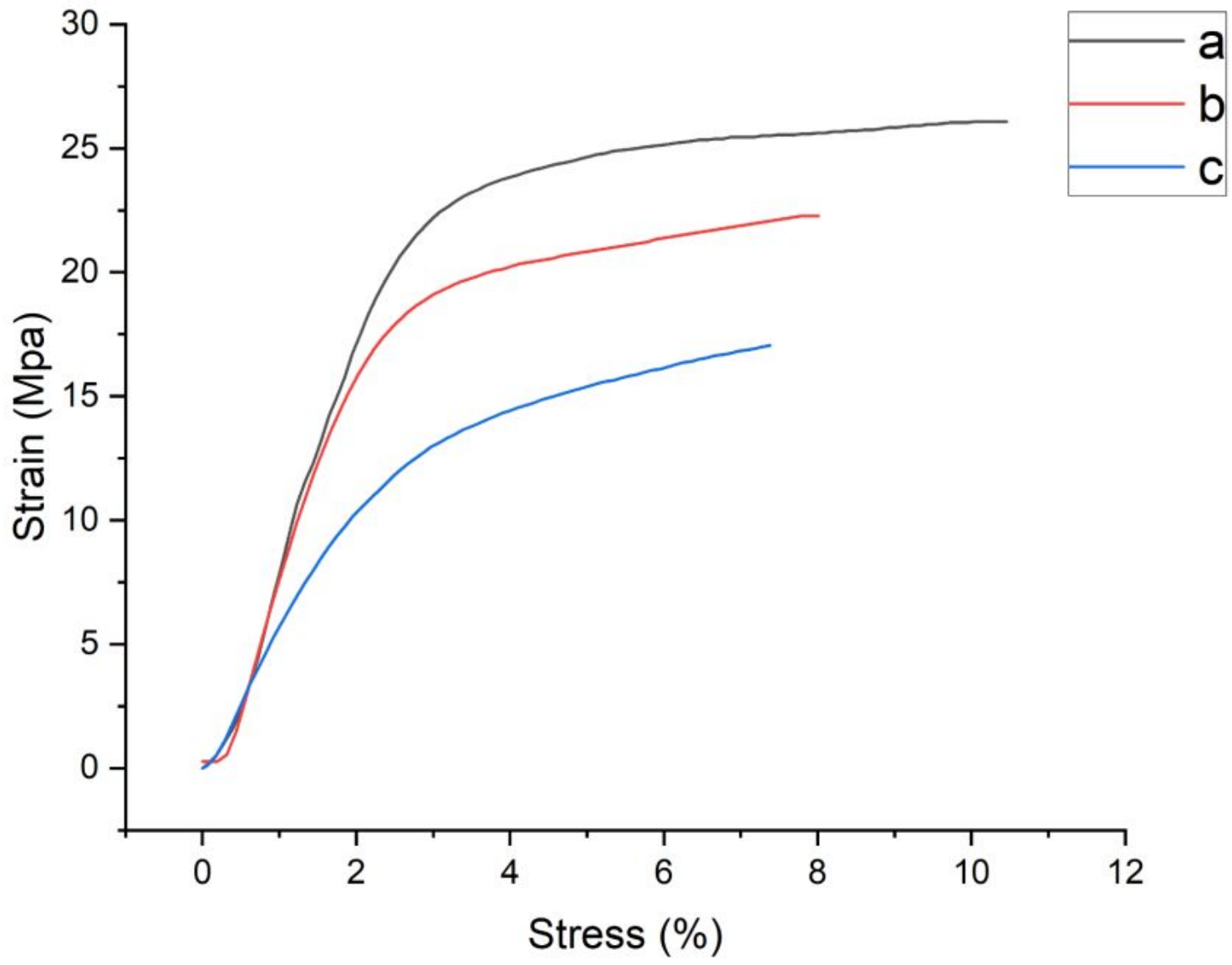

Figure S7. Stress vs. Strain for a) chitosan, b) EHECox4C24, and c) purified EHEC.
